# Integrating proteomics and machine learning reveals characteristics and risks of lymph node-independent distant metastasis in colorectal cancer

**DOI:** 10.3389/fimmu.2025.1622528

**Published:** 2025-07-21

**Authors:** Chenxiao Zheng, Baiwang Zhu, Yanyu Chen, Numan Shahid, Yiwang Hu, Hajar Mansoor Ahmed Ali Husain, Binbin Ou, Qiongying Zhang, Haobo Jin, Yating Zheng, Peng Li, Yifei Pan, Xiaodong Zhang

**Affiliations:** ^1^ Department of Colorectal Anal Surgery, The First Affiliated Hospital of Wenzhou Medical University, Wenzhou, Zhejiang, China; ^2^ Clinical Medical College, Hangzhou Medical College, Hangzhou, Zhejiang, China; ^3^ Department of Hepato-Pancreato-Biliary (HPB) Surgery, King’s College Hospital, Denmark Hill, London, United Kingdom; ^4^ Department of Hepatology, King’s College Hospital, Denmark Hill, London, United Kingdom; ^5^ General Practice Department, Hangzhou Gongshu Hospital of Integrated Traditional and Western Medicine, Hangzhou, Zhejiang, China; ^6^ Department of Pathology, the First Affiliated Hospital of Wenzhou Medical University, Wenzhou, Zhejiang, China; ^7^ Laboratory Animal Centre, Wenzhou Medical University, Wenzhou, Zhejiang, China; ^8^ National Key Clinical Specialty (General Surgery), The First Affiliated Hospital of Wenzhou Medical University, Wenzhou, Zhejiang, China

**Keywords:** colorectal cancer, proteomics, machine learning, synchronous metastasis, immune microenvironment, Itga11

## Abstract

**Background:**

Metastatic colorectal cancer (mCRC) poses significant treatment challenges, especially liver metastasis (CRLM). A notable proportion of CRC has synchronous metastasis independent of lymph node metastasis (LNM). The biological traits of lymph node-independent metastasis in CRC are unclear, and early synchronous metastasis is hard to predict with current imaging or clinicopathological methods.

**Method:**

We collected samples from 12 CRC patients with synchronous distant metastasis without LNM (T1-3N0M1). Data-Independent Acquisition Mass Spectrometry (DIA-MS), multi-omics data integration, and machine learning were used to develop a Lymph node-Independent Metastasis Genes (LIMGs) signature to predict synchronous distant metastasis risk in stage I-II CRC patients and validate it in multi-cohort. Immune microenvironment across risk subgroups was calculated by Estimating Relative Subsets of RNA Transcripts (CIBERSORT). Tumor Mutation Burden (TMB), Microsatellite Instability (MSI) score, immune functions and immune checkpoint gene expression were analyzed to evaluate immunotherapy response. Single cell RNA sequencing (scRNA-seq) analysis illustrated the expression profile of integrin α11 (ITGA11) in CRC. Immunohistochemistry (IHC) confirmed its expression pattern, while wound healing and transwell assays elucidated the role of ITGA11 in CRC metastasis.

**Results:**

The LIMGs signature demonstrated strong predictive performance of lymph node-independent synchronous metastasis across cohorts. The high-risk subgroup exhibited enhanced extracellular matrix (ECM) remodeling, epithelial-mesenchymal transition (EMT) and correlated with immunosuppressive tumor microenvironment (TME), lower TMB and MSI score, indicating worse immunotherapy response. Additionally, machine learning reveal ITGA11’s pivotal role in lymph node-independent metastasis. IHC scores showing significant discriminatory ability of ITGA11 across different samples. Wound healing and transwell assays reveal that the knockdown of ITGA11 hinders the migration and invasion of CRC SW480 cells.

**Conclusion:**

Our findings suggest that EMT-related signature LIMGs significantly affects lymph node-independent distant metastasis in CRC and effectively predicts non-LNM synchronous metastasis in stage I-II CRC patients. LIMG ITGA11 may promote early metastasis by enhancing migration and invasion. These offering insights into precise risk stratification and treatment for CRC patients.

## Introduction

CRC currently ranks as the third most common cancer worldwide and the second leading cause of cancer-related deaths, with over 1,800,000 new cases and nearly 900,000 deaths annually worldwide (1). Metastatic colorectal cancer (mCRC) is one of the challenging aspects in the treatment of CRC, with the liver being the primary site for metastasis (CRLM). Synchronous metastases refer to metastasis detected before or at the time of CRC diagnosis (2). 15%–25% of CRC patients present with distant metastasis at diagnosis, and the vast majority (80%–90%) of CRLM are initially unresectable (3). Liver metastasis is also the leading cause of death in CRC patients, resulting in a significant social burden.

Traditionally, it has been believed that cancer progression involves sequential spread of the tumor to local lymph nodes followed by distant metastasis. However, a considerable number of mCRC patients do not exhibit early systemic spread. Among these, CRLM often occur without lymph node metastasis (LNM). Data indicate that approximately 23% of synchronous liver metastases originate from stage I-II (N0) CRC, and 44% of metachronous metastases arise from N0 CRC (4). A study on resection of CRLM showed that among over 12,000 patients, 37% had no LNM (5). Furthermore, there was no difference in the incidence of liver metastases between patients with and without LNM (6). At the molecular level, CRC metastasis are often proven to originate from a dominant clone within the primary tumor and sharing a high degree of consistency in mutated genes. In contrast, polyclonal origins are more commonly observed in LNM, with 65% of cases showing that LNM and distant metastases arise from independent subclones within the primary tumor (7). Moreover, LNM exhibits a high rate of inconsistency in mutations compared to the primary tumor (8), suggesting lymph nodes may not always be involved in distant metastasis. Animal models have further confirmed that CRC dissemination to the liver can occur independently of LNM, with direct hematogenous spread being a route for CRLM (9). This may imply that stage III and IV CRC may be considered as parallel progression from stage II disease rather than sequential progression.

An incidence model based on tumor size, time, and mutations shows that early metastasis in the majority (80%) of mCRC patients may occur before the primary tumor is clinically detectable (10). As disseminated tumor cells (DTCs) frequently colonize distant organs by the time of primary tumor detection, and they are undetectable with clinical imaging and patients remain asymptomatic regarding subclinical disease. Circulating tumor DNA (ctDNA) and circulating tumor cells (CTCs) show promise as biomarkers for micrometastasis but require enhanced sensitivity and clinical feasibility (11). Effective biomarkers based on tissue-based protein/RNA detection are needed, combining single-cell analysis, detection of ctDNA epigenetic modification, CTC, exosome, immune cell, cytokine may enable real-time predictive biomarker development.

Recent proteomic studies in CRC have revealed novel protein traits, molecular subtypes, and metastasis markers, underscoring molecular heterogeneity across clinicopathological subgroups (12). However, proteomic research on lymph node-independent distant metastasis in CRC remains limited. Epithelial-mesenchymal transition (EMT), which drives early CRC progression by diminishing cell-cell adhesion and apical polarity while enhancing invasion, is of particular interest (13). Here, we hypothesized that lymph node-independent distant metastasis in CRC arises from EMT-related micrometastasis and hematogenous routes. Our study aims to develop a predictive signature for direct distant metastasis risk in early-stage (I-II) CRC by integrating multi-omics data and machine learning, thus refining risk stratification and guiding therapy. To this end, we analyzed 12 synchronous distant metastasis patients (T1-T3N0M1) using DIA-MS. Our findings identify EMT-linked LIMGs as key drivers of lymph node-independent metastasis, with high-risk samples exhibiting a more immunosuppressive tumor microenvironment that may facilitate early distant metastasis.

## Materials and methods

### Patients

**Figure 1:**
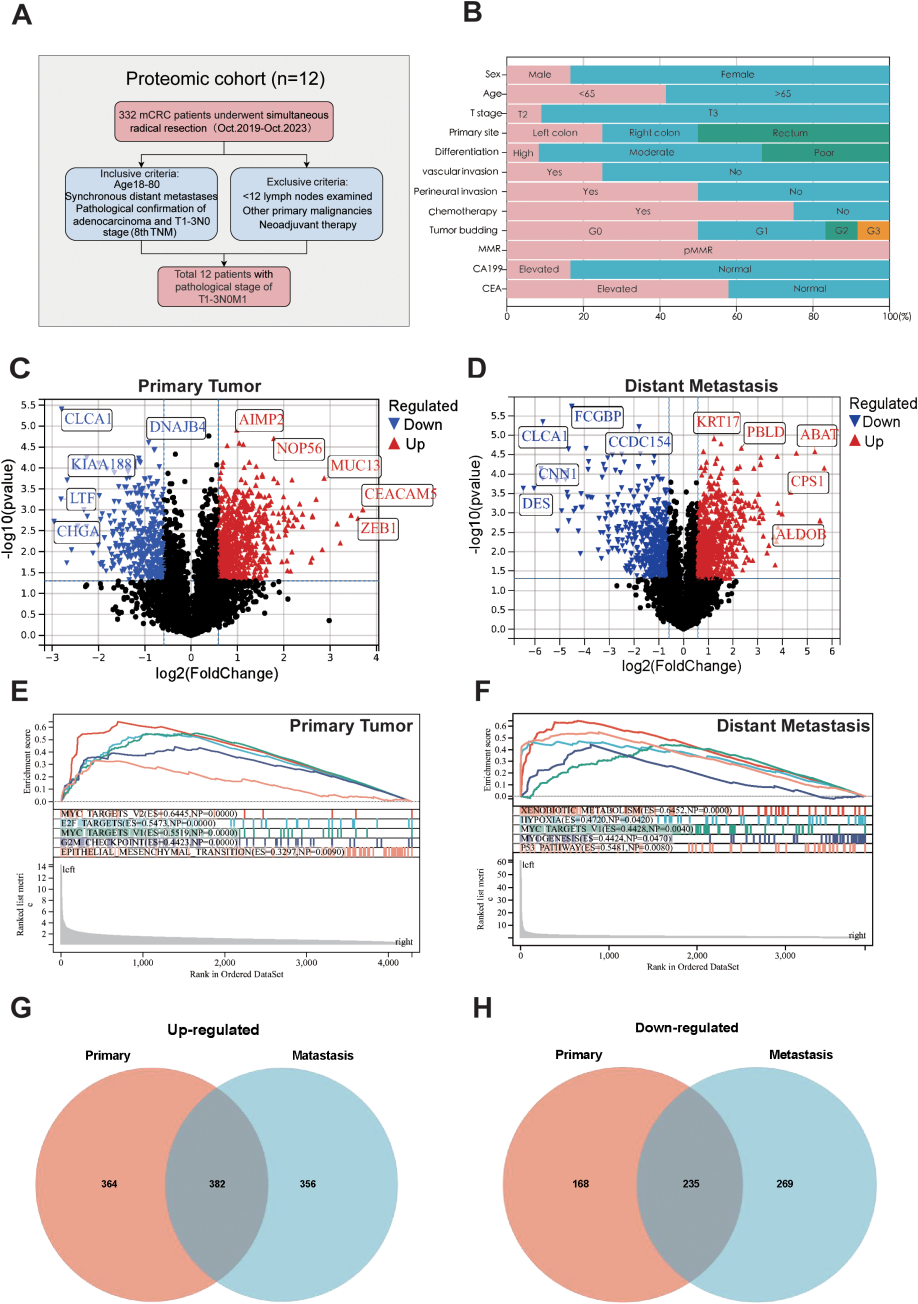
Sample selection and proteomics landscape of T1-T3N0M1 CRC. **(A)** Flow chart of the selection process. **(B)** Clinicopathological parameters are shown in histogram. Volcano plot of the differential expressed proteins in the primary tumors **(C)**, distant metastases **(D)** compared to adjacent normal tissues. GSEA analysis for the differential expressed protein in primary tumors **(E)** and distant metastases **(F)**. Venn plot of up-regulated **(G)** and down-regulated **(H)** proteins in primary tumors and distant metastases.

For the DIA-MS analysis, the patient cohort was sourced from the Colorectal and Anal Surgery Department of the First Affiliated Hospital of Wenzhou Medical University, with the study having secured ethical approval (KY2022-183) from the hospital’s Ethics Committee. Our study screened 271 mCRC patients who underwent simultaneous radical resection of primary tumors and distant metastases between 2018 and 2024. From them, 12 patients with a pathological stage of T1 - 3N0M1 were selected for specimen collection, as shown in **Figure 1A**. The inclusion criteria were age 18 - 80, clinical diagnosis of synchronous distant metastasis, having undergone radical surgery, histopathological confirmation of colorectal adenocarcinoma, and classification as T1 - 3N0M1 stage according to the 8th edition of the AJCC/UICC TNM staging system. Exclusion criteria included lymph node metastasis, an insufficient number of examined lymph nodes (< 12), a history of other primary malignancies, neoadjuvant therapy, and multiple distant metastases. A detailed overview of the clinicopathological characteristics of the study cohort is presented in **Figure 1B** and **Supplementary Table S1**.

### Sample preparation

Formalin-fixed paraffinembedded (FFPE) samples of adjacent normal tissues, primary tumors, and distant metastases were collected from 12 CRC patients. Pathological examination by a pathologist confirmed the tumor areas and using hematoxylin-eosin-stained pathologic slides as reference. All pathological reports were cross diagnosed by two senior pathologists and reviewed by a third. To minimize specimen loss, the same type of tissue sections (4μm) from different patients were prepared and mixed into four composite samples for testing.

### Protein extraction and peptide enzymatic digestion

For protein extraction, each sample was supplemented with an appropriate volume of SDT lysis buffer (4% SDS, 100 mM Tris-HCl, pH 7.6), followed by protein quantification using the BCA method. Subsequently, 15 μg of protein from each sample was mixed with 5× loading buffer, boiled for 5 minutes, and resolved via SDS-PAGE on a 4%–20% precast gradient gel under a constant voltage of 180 **V** for 45 minutes; the gel was stained with Coomassie Brilliant Blue R-250. To generate a quality control (QC) sample, equal amounts of protein from all samples were pooled into a “Pool sample.” All samples, including the QC Pool sample, underwent trypsin digestion using the Filter-Aided Proteome Preparation (FASP) method, after which the resulting peptide fragments were desalted via C18 Cartridge columns, lyophilized, and reconstituted in 40 μL of 0.1% formic acid. Peptide concentrations were determined by measuring absorbance at 280 nm (OD280), and an appropriate quantity of iRT standard peptides was added to each sample prior to analysis by data-independent acquisition (DIA) mass spectrometry using an Astral high-resolution mass spectrometer.

### DIA mass spectrometry analysis

Data-Independent Acquisition Mass Spectrometry (DIA-MS) analysis involved a two-step workflow: (1) chromatographic separation of samples using the Vanquish Neo system (Thermo Fisher) with nanoliter flow rates via nano-HPLC, followed by (2) DIA-MS analysis on the Astral high-resolution mass spectrometer (Thermo Scientific) in positive ion mode (parent ion scan range: 380–980 m/z). First-order mass spectrometry parameters included 240,000 resolutions at 200 m/z, 500% Normalized AGC Target, and 5 ms Maximum Injection Time (IT). DIA data acquisition utilized 299 scan windows (2 m/z isolation window, 25 eV HCD collision energy, 500% Normalized AGC Target, 3 ms IT for MS2). The raw DIA data were processed using DIA-NN software with trypsin digestion (max 1 missed cleavage site), carbamidomethyl (C) as fixed modification, and oxidation (M) and acetyl (N-terminal protein) as dynamic modifications. Database search results were filtered to retain only proteins with a False Discovery Rate (FDR) below 1% (14, 15).

### Data resources

The RNA-seq, proteome datasets and clinical data for CRC patients were obtained from Gene Expression Omnibus (GEO) database, The Cancer Genomic Atlas (TCGA) database (https://portal.gdc.cancer.gov/), and Li et al.’s study cohort CCRC (16), totaling 1,479 samples across GSE39582 (n=585), CCRC (n=146), GSE38832 (n=122), and TCGA-COADREAD (n=626). Differential expression genes (DEGs) were identified using the limma package (Fold change < 0.67 or >1.5, p < 0.05). Overlaps of DEGs in primary tumors and distant metastases were visualized using the “Venn” tool. The CCRC cohort which containing N0M1 CRLM (n=23) and N0M0 (stage I-II, n=49) patients, was used as the training set, validated by GSE39582 and GSE38832, TCGA cohort were utilized for analyzing mutation frequency, TMB, MSI, CNVs and conducting survival analysis, while CRC_EMTAB8107 (n=7) was used for scRNA-seq data analysis.

### Protein-protein interaction network

The STRING database (http://string-db.org/) was employed to explore the interaction relationships among target proteins. Cytoscape software (version 3.10.0) and the GeneMANIA database (http://genemania.org/) were then used to construct a Protein - Protein Interaction (PPI) network, which helped identify the co - expression patterns and interactions of key proteins. By leveraging the Molecular Complex Detection (MCODE, version 2.0.3) plugin (https://apps.cytoscape.org/apps/mcode), we extracted potentially densely interconnected gene modules from the PPI network.

### Biological function and pathway enrichment analysis

To unravel the biological functions and pathways associated with differentially expressed genes (DEGs) and the core cluster within the PPI network, we utilized the “ClusterProfiler” package and Gene Set Enrichment Analysis (GSEA) software, which can be accessed at https://www.gsea-msigdb.org/gsea/index.jsp. With these tools, we performed Kyoto Encyclopedia of Genes and Genomes (KEGG), Gene Ontology (GO), and GSEA analyses. To evaluate the correlation between gene expression levels and biological pathways or molecular mechanisms, we downloaded the h.all.v7.4.symbols.gmt subset from the Molecular Signatures Database (MSigDB), available at https://www.gsea-msigdb.org.

### Machine learning identifies LIMGs prognostic biomarkers

Wilcoxon test identified differentially expressed genes between N0M0 (stage I-II) and N0M1 patients, Lasso regression eliminated redundant genes through ten-fold cross-validation using the glmnet package (17). Logistic analysis was used after Z-score transforming of the expression data to determine the odds ratio (OR) of potential hub genes and understand their contribution to the metastasis. Finally, 9 genes were identified as LIMGs. The diagnostic performance of LIMGs was validated using ten machine learning algorithms including Logistic, Support Vector Machine (SVM), Gradient Boosting Machine (GBM), Neural Network, Random Forest (RSF), XGboost, K-Nearest Neighbors (KNN), Adaptive Boosting (Adaboost), Light Gradient Boosting Machine (Light GBM), and Categorical Boosting (CatBoost). We applied them to the CCRC cohort for training, and further validated on external datasets (GSE39582, GSE38832), ROC curves generated by the pROC package were utilized to evaluate the accuracy of the model in diagnosing lymph node-independent distant metastasis of I-II stage CRC. For each patient, LIMG score was calculated for each sample and stratified them into subgroups based on the median score, Kaplan-Meier (KM) survival analysis and nomogram (rms package) assessed prognostic significance of LIMGs.

### Mutation analysis and immune microenvironment

Based on the LIMGs Score, risk subgroups are classified in TCGA-COADREAD cohort. Utilizing the “mafTools” R package we analyzed the differences in somatic mutations, TMB between high-risk and low-risk groups, as well as mutation frequency in 9 LIMGs across all samples. The total count of non-synonymous somatic mutations per megabase across the entire genome was computed to assess the TMB. The CNV data and MSI score of CRC patients were downloaded using the TCGA bio links package. Using the CIBERSORT algorithm (18), we evaluated the abundance of 24 immune cell subsets in different risk subgroups. The immune-related functions and expression differences of immune checkpoint genes between subgroups calculated by ssGSEA package predicted immunotherapy response. Correlations between ITGA11 and immune cells were calculated using TIMER (19), QUANTISEQ (20), MCPcounter (21), EPIC (22), and CIBERSORT (23).

### Drug sensitivity analysis

Based on Cancer Therapeutics Response Portal (CTRP, https://portals.broadinstitute.org/ctrp.v2.1/) and Genomics of Drug Sensitivity in Cancer (GDSC, https://www.cancerrxgene), the “Oncopredict” R package was used to conduct a half-maximal inhibitory concentration (IC50) analysis of drugs for high-risk and low-risk groups of CRC patients.

### Single cell RNA sequencing analysis

We acquired a CRC dataset (CRC_EMTAB8107) from the Tumor Immune Single Cell Hub 2.0 (TISCH 2.0) database (http://tisch.compgenomics.org/) (24), comprising 23,176 cells from 7 tumor samples. Subsequent analyses included scRNA-seq for the ITGA11 and Cell-Cell Interaction (CCI) analysis and visualize the expression and distribution of ITGA11, and the interactions between target gene-enriched cell subpopulations and others.

### Antibodies, plasmids, cell lines and culture

In this study, two antibodies were utilized: ITGA11 (#DF8992, Affinity Biosciences, USA) and GAPDH (#2118, Cell Signaling Technology, USA). The SW480 cell line (#CBP60019), authenticated by short tandem repeat (STR) profiling, was procured from the Chinese Academy of Sciences (CAS). Cells were cultured in RPMI 1640 medium (#C11875500BT, Gibco, USA) supplemented with 10% fetal bovine serum (FBS) and 10,000 U/ml penicillin-streptomycin (#15140122, Gibco, USA) in a humidified incubator with 5% CO_2_. A knockdown plasmid targeting ITGA11 was synthesized by Miaoling Bioscience (Wuhan, China).

### Immunohistochemical assay

Tissue specimens were fixed in 4% paraformaldehyde, embedded in paraffin, and sectioned into 4 µm-thick slices for slide preparation. After gradient deparaffinization and rehydration, antigen retrieval was performed using a microwave method with citrate buffer (100°C, four cycles of 7 minutes each). The slides were washed extensively with PBS and then blocked for 30 minutes to minimize nonspecific binding. The primary antibody was incubated overnight at 4°C, followed by incubation with the secondary antibody at room temperature. Color development was achieved using DAB chromogen, and the sections were counterstained with hematoxylin.

### Wound healing assay

Cells were plated in 6-well plates and grown to confluence. A sterile pipette tip scratched the monolayer, which was then washed with PBS to remove any dislodged cells. Culture medium with 1% (fetal bovine serum) FBS was added. Images of cell migration were taken at 0, 24, and 48 hours post-wounding. The wound closure area was calculated as: Migration Area (%) = (X0 - Xn)**/**X0 × 100, where X0 is the initial wound area and Xn is the area at a specific time.

### Transwell assay

The invasive and metastatic potential of SW480 cells was assessed using a Matrigel-coated Transwell assay. Briefly, 3×10^4 cells were seeded in the upper chamber of a Transwell with serum-free medium, while the lower chamber contained 10% FBS-supplemented medium. After 24 hours of incubation at 37°C, cells in the upper chamber were fixed with methanol and stained with Giemsa for quantitative microscopic analysis of invasion and migration.

### Western blot assay

Cell proteins were extracted using a lysis buffer (10 mM TRIS-HCl, pH 7.4, 1% SDS, 1 mM Na_3_VO_4_) and lysed via ultrasonic treatment. Protein concentration was quantified using a microspectrophotometer. Samples, mixed with loading buffer and a molecular weight marker, were loaded onto an 8% SDS-PAGE gel and subjected to electrophoresis at 80 **V** for 30 minutes, followed by 120 **V** for 90 minutes. Proteins were transferred to a PVDF membrane (25 **V**, 120 minutes) and blocked in buffer at 4°C for 3 hours. The membrane was then incubated overnight with primary antibodies at 4°C and for 3 hours with secondary antibodies. Protein bands were visualized using an ECF developer (RPN5785, GE Healthcare) and captured using a chemiluminescent imaging system (GE Healthcare).

### Statistical analysis

All statistical analyses were conducted using R software (version 4.4.2). The Wilcoxon test compared variables between groups, the Chi-square test assessed categorical variable differences, Pearson correlation analyzed variable correlations, and KM survival analysis with log-rank test evaluated differences. Statistical significance was set at p<0.05: *: p<0.05; **: p<0.01; ***: p<0.0001; NS: non-significant.

### Ethics approval

This study was conducted in accordance with the ethical standards outlined in the Helsinki Declaration and certified by the Ethics Committee of The First Affiliated Hospital of Wenzhou Medical University (KY2022-183). Given the retrospective nature of the study, informed consent was waived.

## Results

### Proteomic characteristics of T1-T3N0M1 patients

To identify the protein signatures and pathways associated with T1-T3N0M1 CRC, we used adjacent normal tissue as a control and analyzed the differentially expressed proteins in primary tumor and distant metastasis and conducted a comprehensive comparison of biological pathways and functions. Differential analysis revealed 746 upregulated and 403 downregulated proteins in primary tumors (**Figure 1C**), and 751 upregulated and 321 downregulated in distant metastases (**Figure 1D**). GSEA was performed to analyze the features of the proteins detected in primary tumors and distant metastases in terms of biological pathways and molecular mechanisms. Results showed enrichment in MYC targets V2, E2F targets, MYC targets V1, G2M Checkpoints, EMT pathway in primary tumors (**Figure 1E**). Xenobiotic metabolism, Hpoxia, MYC targets V1, Myogenesis, P53 pathway enrichment in distant metastases (**Figure 1F**). Analysis of GO and KEGG pathway of these proteins is provided in **Supplementary Figure S1**. Venn diagrams visualized the intersections of differentially expressed proteins among primary tumors and metastases (**Figures 1G, H**).

### Construction of PPI networks and module identification for biomarker discovery

**Figure 2:**
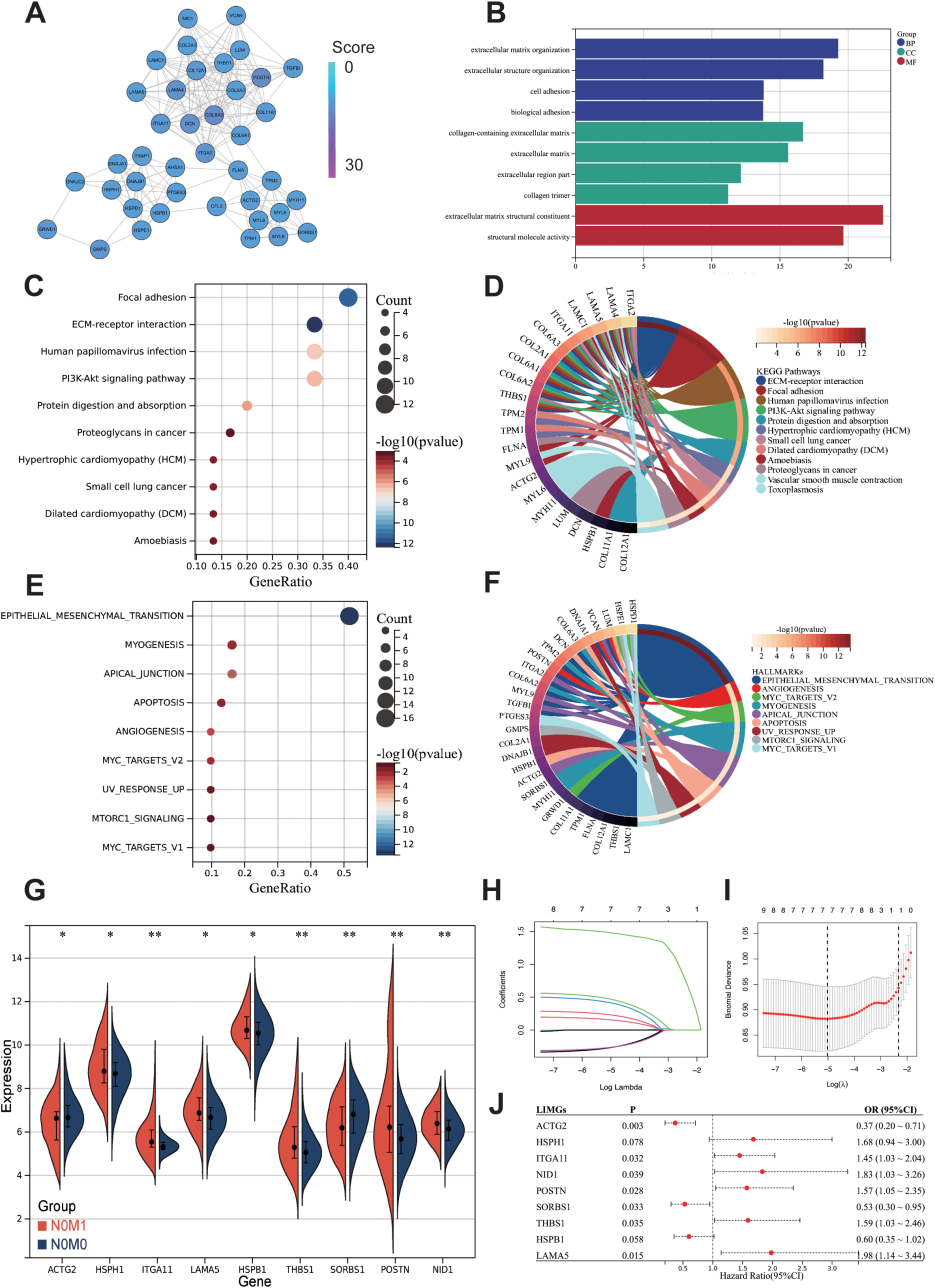
Preliminary screening of core biomarkers associated with lymph-node independent metastasis. **(A)** MCODE in Cytoscape identified a module consisting of 40 nodes from the PPI network. The GO **(B)**, KEGG **(C)** and Hallmark gene sets **(E)** enrichment analysis of the 40 genes from cluster2. Chord diagrams of KEGG **(D)**, and Hallmark gene sets **(F)** enrichments show associations of 40 genes across different biological aspects. Differentially expressed genes between N0M0 and N0M1 **(G)**. The Lasso regression path plot **(H)** and cross-validation plot **(I)** illustrate the gene selection process. The univariate logistic regression results of LIMGs **(J)**. Statistical signifificance: p<0.05; **: p<0.01; ***: p<0.0001; NS: non-signifificant.

To construct a PPI network for biomarker identification, we uploaded 617 differentially expressed proteins shared between primary tumors and distant metastases into the STRING database. The resulting network was visualized using Cytoscape software and the MCODE plugin, enabling the identification of the five most functionally significant modules (**Supplementary Figure S2**). Among these, Cluster 2 emerged as a key module, comprising 40 nodes and 214 edges (**Figure 2A**). GO analysis reveals that Cluster2 is mainly enriched in ECM organization, cell adhesion, collagen-containing ECM, and ECM structural constituent (**Figure 2B**). The KEGG analysis indicates the enrichment of Focal adhesion, ECM-receptor interaction, and PI3K-Akt signaling pathway (**Figures 2C, D**). Hallmark pathway enrichment analysis shows enrichment in EMT pathway, Myogenesis, Apical junction, Apoptosis, and Angiogenesis (**Figures 2E, F**).

### Machine learning identifying LIMGs signature and constructing diagnostic model

**Figure 3:**
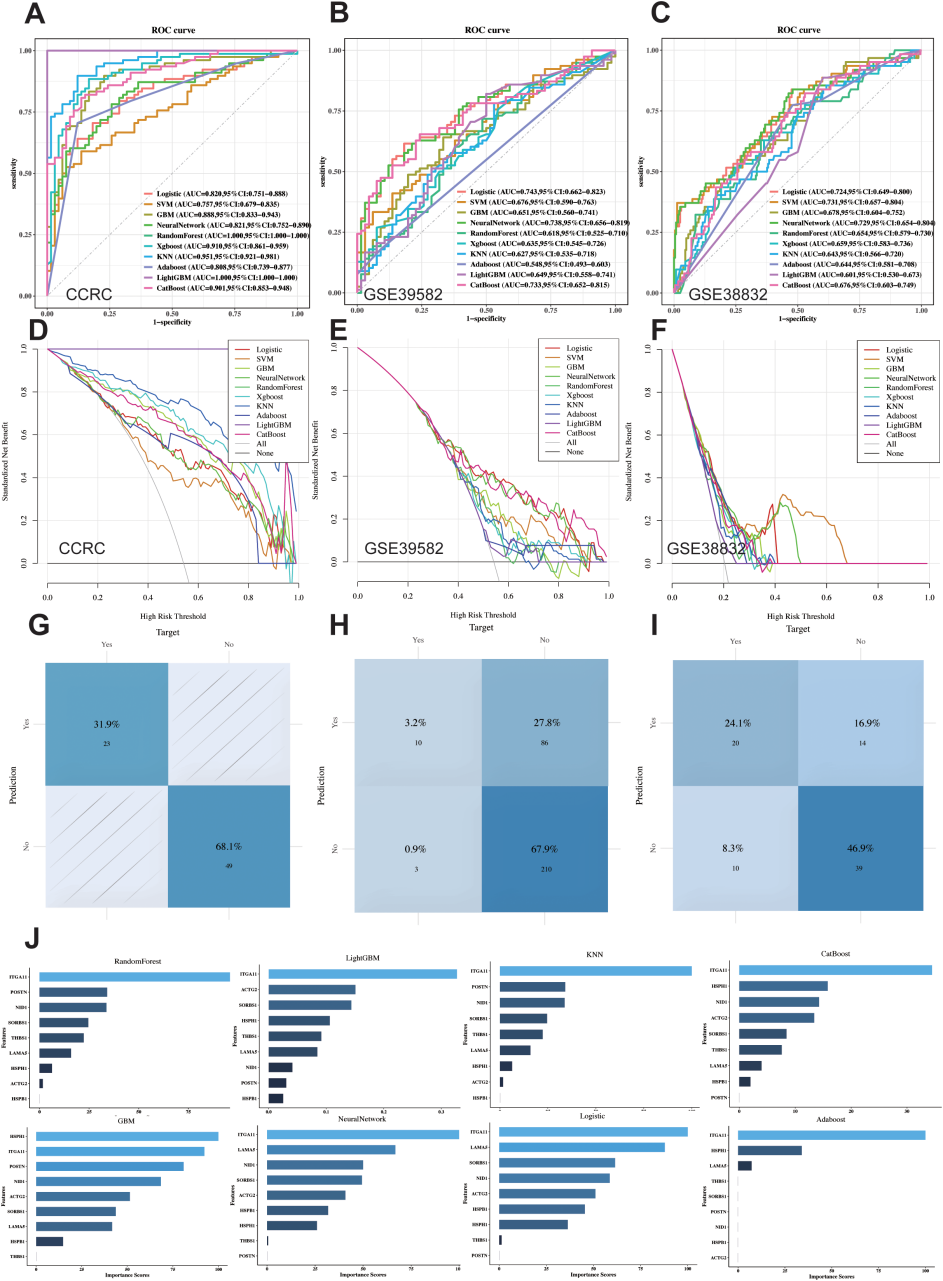
Ten Machine learning methods assess the diagnostic performance of LIMGs signature. ROC curves of ten machine learning methods (Logistic, SVM, GBM, Neural Network, RF, XGboost, KNN, Adaboost, Light GBM, and CatBoost) applied in CCRC training cohort **(A)** and external validation cohort GSE39582 **(B)** and GSE38832 **(C)**. Cost-benefit decision curves in training **(D)** and validation **(E, F)** cohorts. Classification confusion matrix of the Neural Network model in training **(G)** and validation cohort **(H, I)**. **(J)** The feature importance bar chart illustrates variable contributions to the top 8 models in training cohort.

To further identify core biomarkers and establish an accurate diagnostic model, we identified differentially expressed genes in Cluster2 between stage I-II and N0M1 CRC (Wilcoxon test, p<0.05) (**Figure 2G**), after eliminating redundant genes using Lasso regression (**Figures 2H, I**), 9 genes were selected as LIMGs (ACTG2, HSPH1, ITGA11, LAMA5, HSPB1, THBS1, SORBS1, POSTN, NID1). Univariate logistic regression highlighted the importance of LIMGs via OR (**Figure 2J**). Ten machine learning algorithms including Logistic, SVM, GBM, Neural Network, Random Forest, XGboost, KNN, Adaboost, Light GBM, and CatBoost were then applied to assess the diagnostic efficacy of LIMGs in the CCRC training set, ROC curves (**Figure 3A**), DCA (**Figure 3D**), confirmed robust diagnostic performance, with external validation in two cohorts (GSE39582, **Figures 3B, E**), (GSE38832, **Figures 3C, F**). The Neural Network model demonstrated consistent performance across cohorts, with diagnostic efficacy displayed by the confusion matrix (**Figures 3G–I**). Feature importance analysis of the top eight models in the training cohort identified ITGA11 as the key factor influencing lymph node-independent metastasis (**Figure 3J**).

### LIMGs correlate with poor prognosis and clinicopathological features

**Figure 4:**
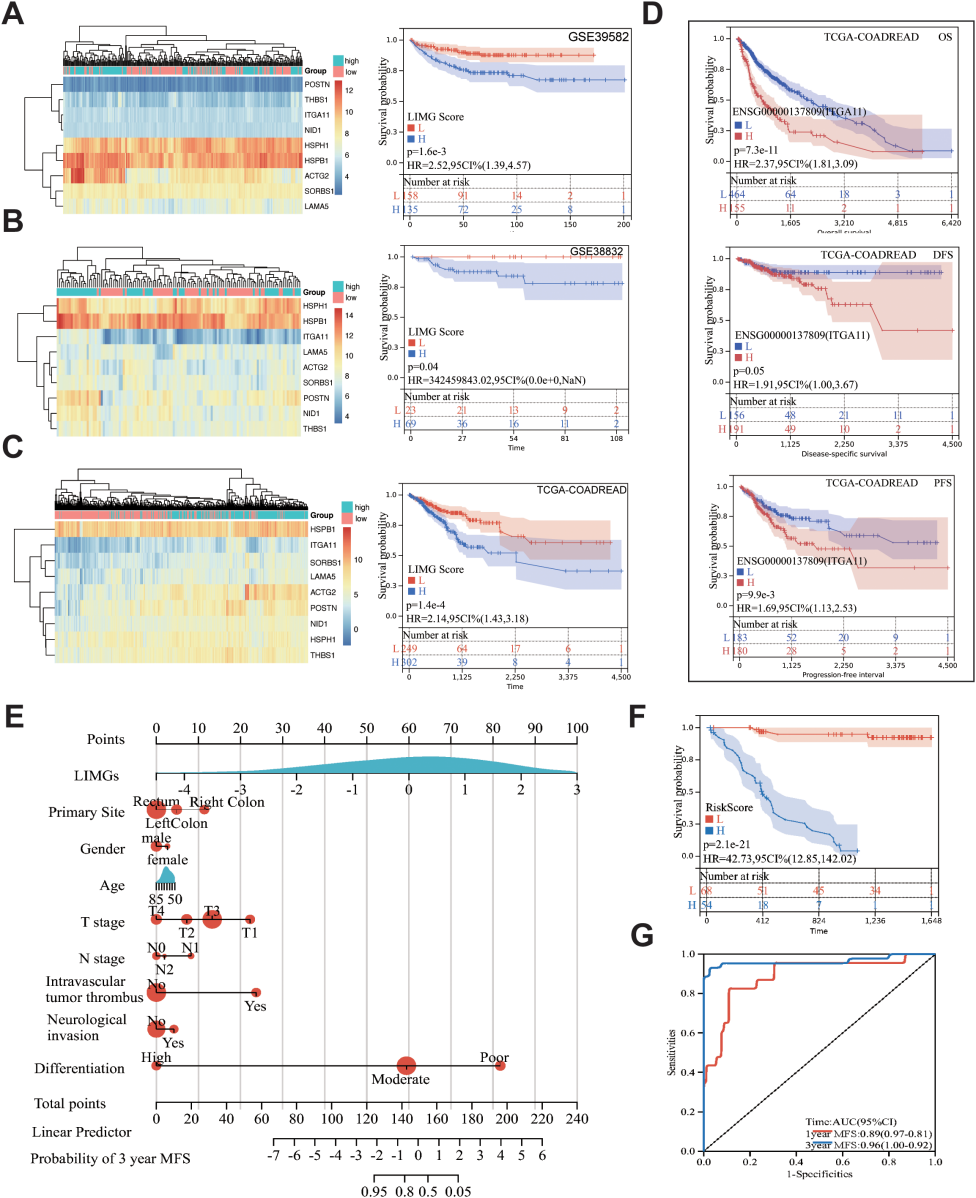
The correlation between the LIMGs and survival. Heatmaps of LIMGs expression and the KM curves stratified by high-risk and low-risk groups based on median LIMG Score in the GSE39582 **(A)**, GSE38832 **(B)**, and TCGA cohorts **(C)**. **(D)** KM curves for overall survival (OS), disease-free survival (DFS), and progression-free interval (PFI) in high-risk and low-risk groups stratified by the optimal cutoff value of ITGA11 expression in TCGA-COADREAD. **(E)** Nomogram for predicting Metastasis-free survival (MFS) was constructed using multivariate Cox regression. **(F)** KM curves compared high-risk vs. low-risk stratified by median risk score. **(G)** ROC curves evaluated 1-year/3-year MFS prediction accuracy of the Nomogram.

**Figure 5:**
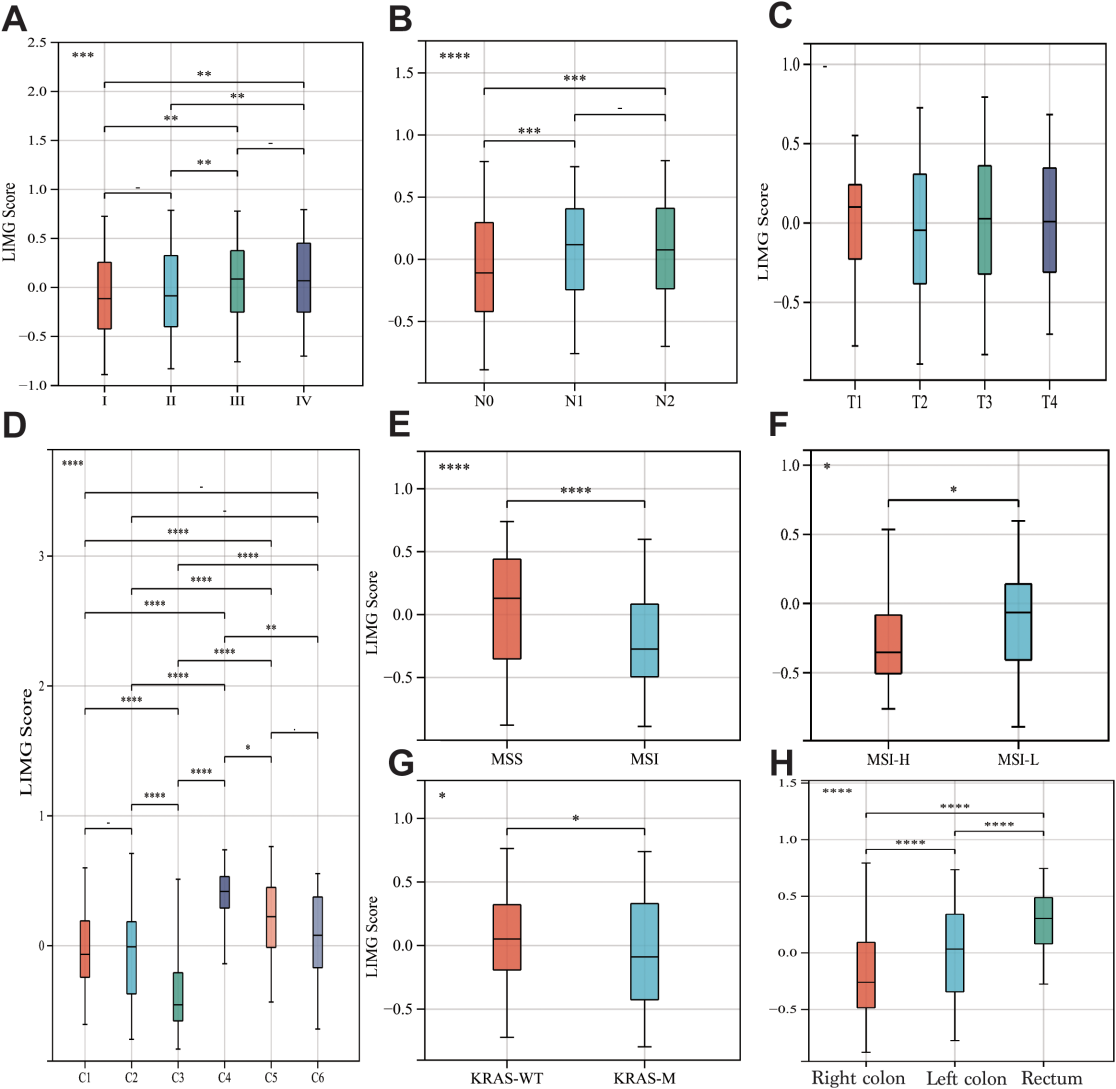
The correlation between LIMGs and clinicopathological features. The correlation between the LIMG Score and AJCC stage **(A)**, N stage **(B)**, T stage **(C)**, Molecular subtypes **(D)**, Microsatellite stability **(E, F)**, KRAS mutation status **(G)**, and tumor location **(H)**. Statistical signifificance: *: p<0.05; **: p<0.01; ***: p<0.001; ****: p<0.0001; NS, non-signifificant.

The GSVA method scored GSE39582 (**Figure 4A**), GSE38832 (**Figure 4B**) and TCGA-COADREAD (**Figure 4C**) samples based on LIMGs expression, classifying risk subgroups by the median score. KM curves revealed significant survival difference between risk subgroups. KM curves for subgroups based on ITGA11 median expression revealed its significant impact on overall survival (OS), Disease-free survival (DFS), and Progression-free interval (PFI) (**Figure 4D**). To further explore the association between LIMGs and metastasis of patients, we employed the R package rms to integrate data on metastasis-free survival (MFS), survival status, and eight relevant features of CCRC cohort. A nomogram was constructed using the Cox method, and the prognostic significance of these features was assessed in 143 samples of CCRC cohort (**Figure 4E**). Kaplan-Meier curves (**Figure 4F**) and ROC curves for 1- and 3-years MFS (**Figure 4G**) underscored the predictive accuracy of LIMGs, highlighting its value in predicting metastasis. Further evaluation for the association between the LIMGs and other pathological characteristics reveals that higher LIMG score is significant associated with advance AJCC stage (**Figure 5A**), N stages, (**Figure 5B**), MSI status (**Figures 5E, F**), KRAS-WT (**Figure 5G**), and the left-sided colorectal cancer (**Figure 5H**) (all p<0.05). Furthermore, CRC is molecularly classifed into six subtypes by Marisa et al. (25) including C1 (downregulation of immune pathway), C2 (MSI subtype), C3 (KRAS mutant), C4 (chromosomal instability and stem-like), C5 (Wnt pathway upregulation) and C6 (derived from serrated tumors). We found that higer LIMG score correlated with C4-C6 molecular subtypes (**Figure 5D**). Additionally, no significant differences in LIMG score were observed across different T stages (**Figure 5C**), ages, sexes, vascular invasion statuses, histological types, and BRAF, TP53 mutation statuses (**Supplementary Figure S3**).

### Analysis of LIMGs interaction and correlation with EMT

**Figure 6:**
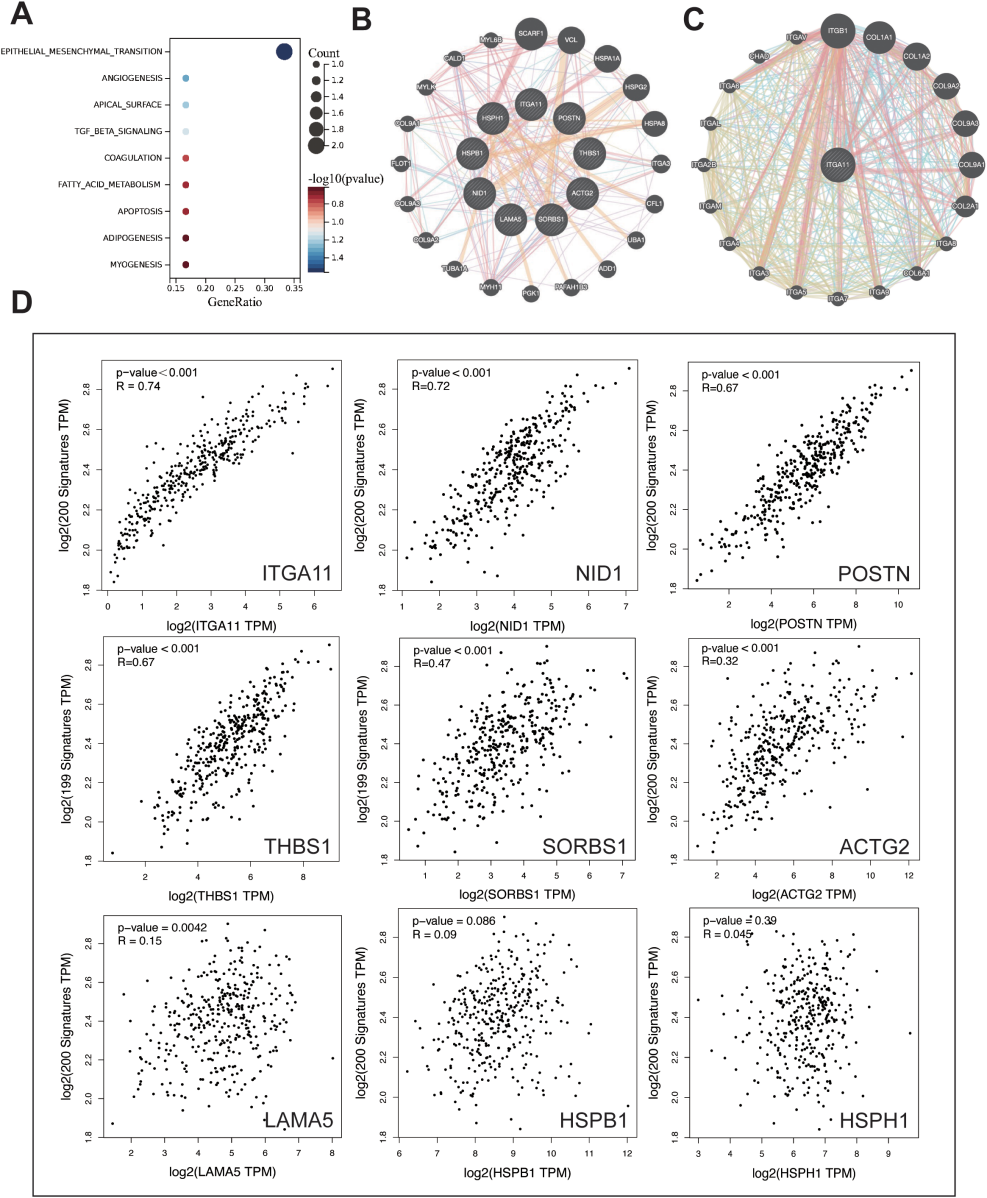
The enrichment and interaction analysis of LIMGs and correlation analysis between LIMGs expression and the EMT pathway. **(A)** Hallmark enrichment analysis plot of LIMGs. **(B)** Interactions among LIMGs. **(C)** The PPI network centered on ITGA11. **(D)** Correlation between LIMGs expression and the EMT pathway gene set.

The hallmark enrichment revealed that LIMGs are mainly enriched in the EMT pathway (**Figure 6A**). EMT drives tumor invasion and metastasis through induction of stemness, modulation of the TME, angiogenesis promotion, and metabolic reprogramming. We investigated the correlation between LIMGs and the EMT pathway by 200 EMT-related genes from the MSigDB database v7.1. The correlation between LIMGs and EMT gene signatures was analyzed using Pearson correlation analysis on the GEPIA2 (http://gepia2.cancer-pku.cn/#index). The results indicated that ITGA11 has the strongest correlation with EMT (**Figure 6D**). Then we used GeneMANIA to analyze the interactions among LIMGs (**Figure 6B**) and the PPI network centered on ITGA11 (**Figure 6C**). The results of GO and KEGG enrichment analyses of LIMGs are shown in **Supplementary Figure S4**.

### Mutation landscape and immune activity in different risk groups

**Figure 7:**
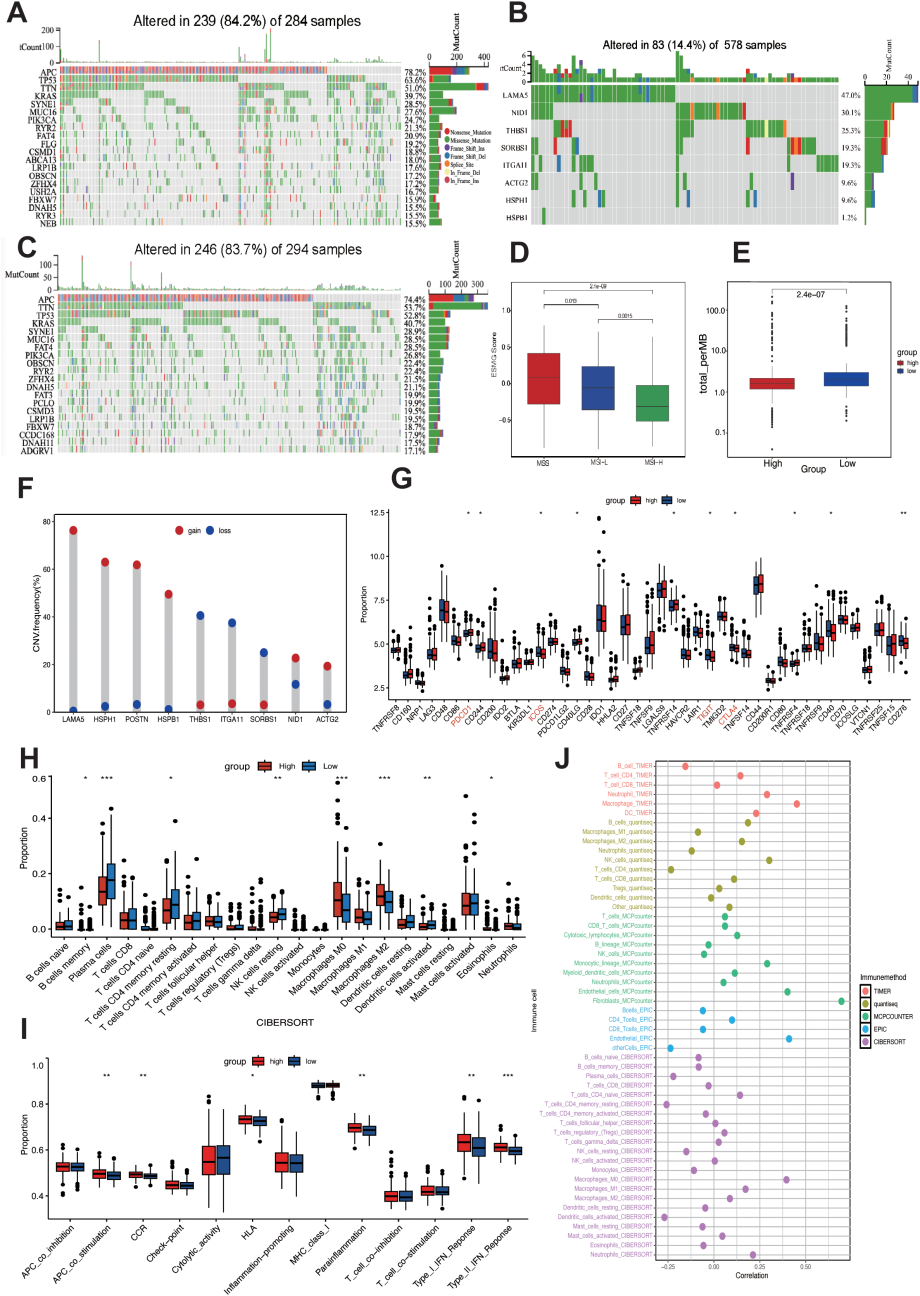
Mutation landscape and immune activity analysis. Top 20 mutated genes in high-risk **(A)** and low-risk **(C)** subgroups. **(B)** Mutation frequency of 9 LIMGs. **(D)** The LIMG Score of CRC patients with microsatellite instability-high (MSI-H), microsatellite instability-low (MSI-L) and microsatellite stability (MSS). **(E)** Comparison of TMB in high- and low- risk subgroups. **(F)** The CNV frequency of each LIMG signature genes. **(G)** Differentially expressed immunocheckpoint genes across risk subgroups. **(H)** Differences in immune cell infiltration across risk subgroups. **(I)** Immune-related functions in the high- and low- risk subgroups. **(J)** Correlation between ITGA11 expression and immune cells. Statistical signifificance: *:p<0.05; **: p<0.01; ***: p<0.0001; NS: non-signifificant.

To elucidate the distinct mutational patterns among different risk groups, we utilized the “mafTools” R package to analyze the distribution of top 20 somatic mutations between risk groups and mutation status in 9 LIMGs based on TCGA-COADREAD data. Our findings revealed that APC, TP53, TTN, and KRAS exhibited high mutation frequencies across different subgroups, with APC being identified as the most frequently mutated gene across subgroups (**Figures 7A, C**). LAMA5 showed the highest mutation frequencies among LIMGs (**Figure 7B**). Meanwhile, copy number variation (CNV) plays a crucial role in cancer occurrence and development. We found that the highest CNV in LIMGs was also found in LAMA5 (**Figure 7F**). Given the significance of TMB, MSI status, immune cell infiltration, immune functions, and immune checkpoint gene expression in immunotherapy response, we examined their relationship with LIMGs. Immune infiltration analysis by CIBERSORT revealed that the high-risk group had lower proportions of memory B cells, plasma cells, CD4+ T cells, NK cells, dentritic cells and eosinophils but higher proportions of M0 and M2 macrophages (**Figure 7H**). Moreover, the high-risk group exhibited greater immunological function, including higher levels of Type I and II IFN Response and APC co-stimulation. (**Figure 7I**). The high-risk group also exhibited a significantly lower MSI proportion (**Figure 7D**) and lower TMB (**Figure 7E**). Conversely, the TMB and MSI status was higher in low-risk group, suggesting better immunotherapy response. Analysis of immune checkpoint expression showed higher expression of PDCD1 in low-risk group and higher expression of TIGIT, ICOS and CTLA4 in high-risk group (**Figure 7G**). Furthermore, the positive correlation between ITGA11 expression and various immune cells was calculated by five algorithms (TIMER, QUANTISEQ, MCPcounter, EPIC, CIBERSORT) (**Figure 7J**), specifically with high levels of CAFs and TAMs. This correlation may indicate poor prognosis in CRC patients with higher level of ITGA11+CAFs and ITGA11+TAMs. In summary, the high-risk group exhibited lower TMB, MSI status and immunosuppressive TME, suggesting less favorable immunotherapy outcomes compared to the low-risk group.

### LIMGs associate with lower chemotherapy sensitivity

**Figure 8:**
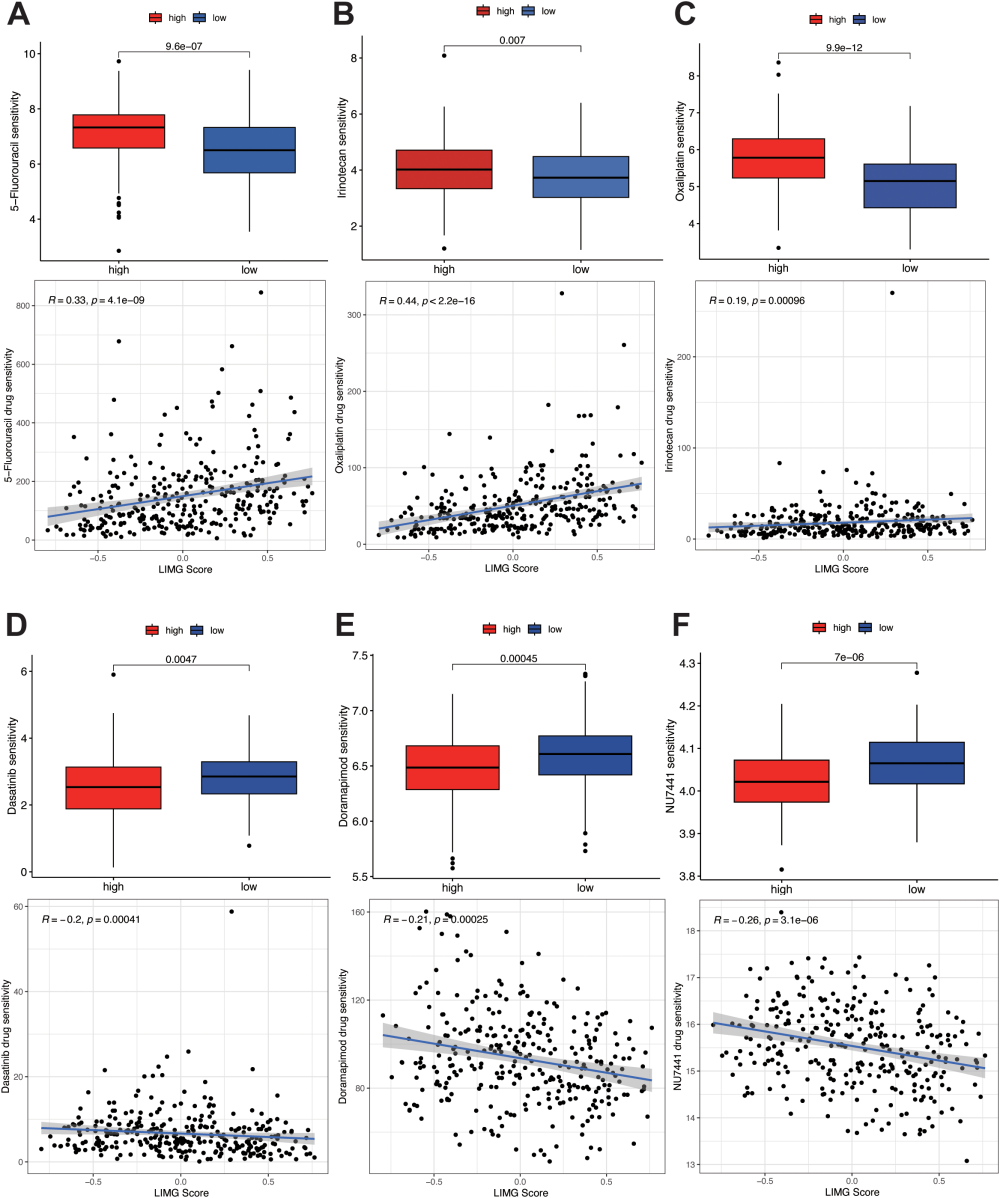
Drug sensitivity analysis. Sensitivity analysis of 5-fluorouracil **(A)**, oxaliplatin **(B)**, and irinotecan **(C)** in different risk groups. Sensitivity analysis of Dasatinib **(D)**, Doramapimod **(E)**, and PRKDC inhibitor NU7441 **(F)** in different risk groups.

To predict drug sensitivity and identify potential therapeutic drugs for high-risk CRC patients, we calculated IC50 values for three commonly used CRC chemotherapy drugs (5-Fluorouracil, Oxaliplatin, Irinotecan) in different risk subgroups and assess the correlation between LIMG score and drug sensitivity. The results showed that high-risk patients had poorer sensitivity to 5-Fluorouracil (**Figure 8A**), Oxaliplatin (**Figure 8B**), and Irinotecan (**Figure 8C**), with IC50 values positively correlated with risk scores. Conversely, high risk patients exhibited higher sensitivity to Dasatinib (**Figure 8D**), Doramapimod (**Figure 8E**), and PRKDC inhibitor NU7441 (**Figure 8F**), with IC50 values negatively correlated with LIMG score.

### ScRNA-seq analysis of ITGA11

**Figure 9:**
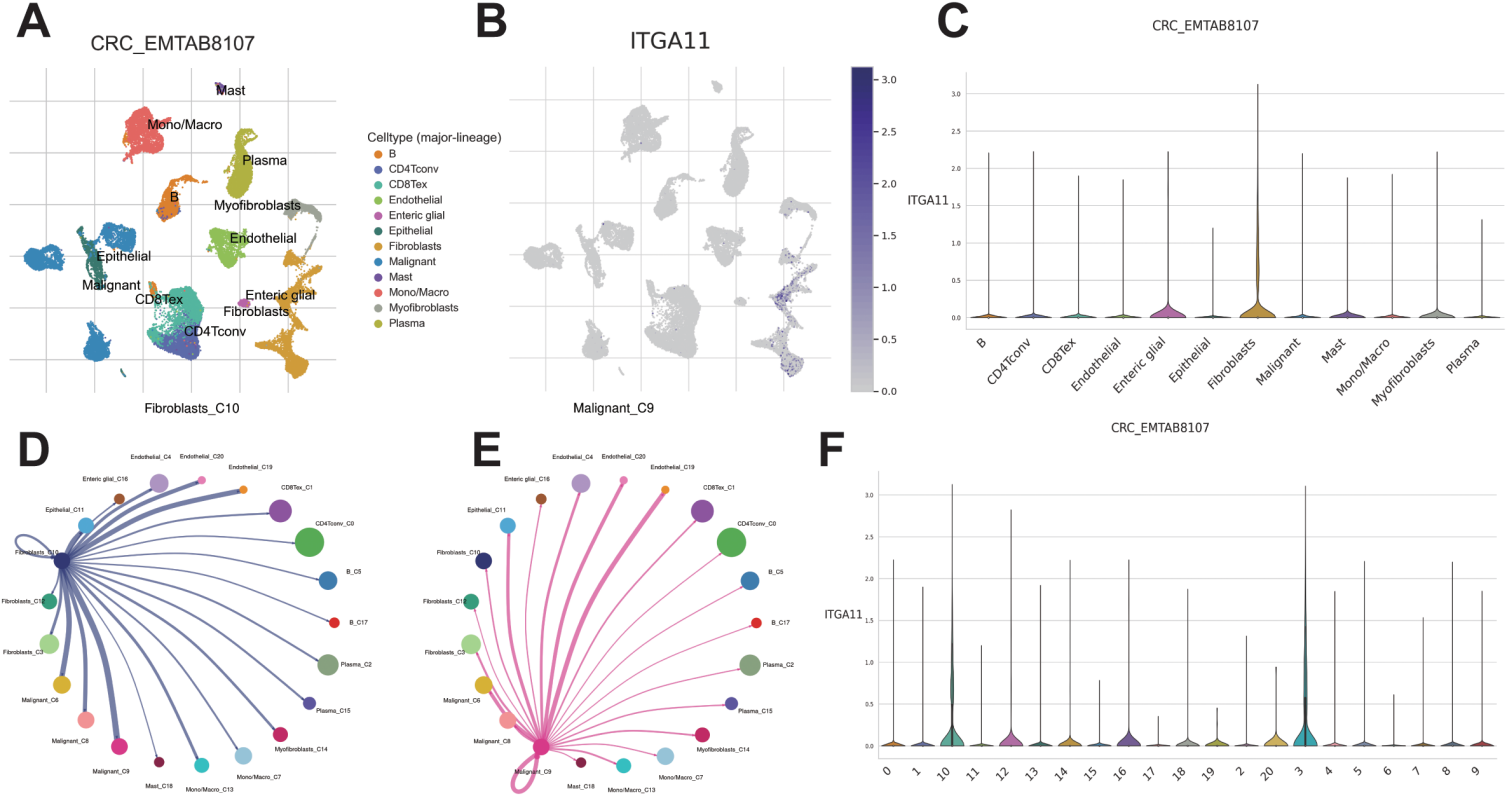
Single cell RNA sequencing analysis. **(A)** UMAP projection of all cells from CRC_EMTAB8107. **(B, C)** Expression distribution of ITGA11 across different cell types. CCI analysis between endothelial cluster C_4 **(D)** and fibroblast cluster C_12 **(E)**. **(F)** Expression distribution of ITGA11 across different cell clusters.

To explore the expression and distribution of ITGA11 in the TME at the single-cell level, we conducted scRNA-seq analysis using the TISCH 2.0 database. Analysis of scRNA-seq data from the CRC_EMTAB8107 dataset revealed the identification of 20 cell clusters and 12 cell types within CRC tissues (**Figure 9A**). We observed a significant enrichment of ITGA11 in CAFs (**Figure 9B**), especially within clusters C3 and C10 (**Figure 9F**). The analysis of Cell-Cell Interactions (CCI) revealed that both C3 and C10 CAFs mainly interacted with C9 malignant cells and C19 endothelial cells (**Figures 9D, E**).

### ITGA11 promotes migration and invasion of colorectal cancer cells

**Figure 10:**
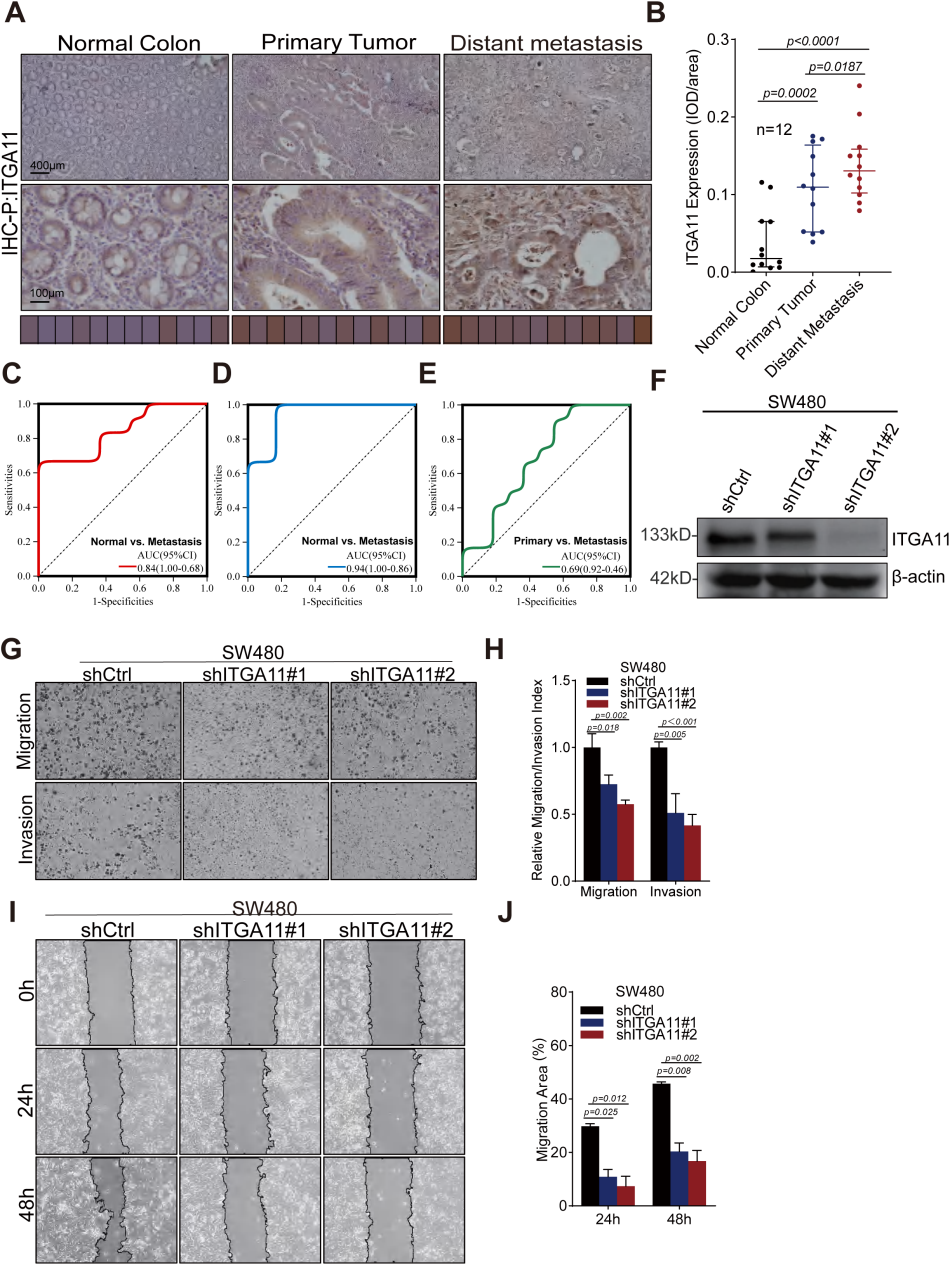
ITGA11 expression variation across tissues and its role in the migration and invasion of CRC cell. **(A)** Immunohistochemical analysis of ITGA11 expression in normal tissues, primary tumors, and distant metastases. **(B)** The Wilcoxon test indicated a significant difference (P<0.05) in ITGA11 expression across normal tissues, primary tumors, and distant metastases. **(C–E)** The ROC curves indicated that ITGA11 expression effectively differentiated normal tissues, primary tumors, and distant metastases. **(F)** The ITGA11 expression in human colon cancer cells SW480 was measured by western blotting. **(G, H)** Results of the transwell assay. **(I, J)** Results of the wound healing assay.

In this study, we unveiled the crucial role of LIMGs in distant metastasis of CRC, primarily associated with cell adhesion and EMT. Notably, LIMG ITGA11 is the gene most strongly correlated with EMT. Although ITGA11 overexpression has been reported in several tumors, its impact on CRC cell migration and invasion remains unexplored. The radar chart illustrates the expression levels of LIMGs (Log2(FC)) based on our proteomics data (**Supplementary Figure S5**). The results reveal the expression across primary tumors, and distant metastasis for 9 LIMGs. Among them, ITGA11 showed higher expression in distant metastasis than in primary tumors. The IHC score further confirmed higher ITGA11 expression in both primary tumors and distant metastasis compared to normal tissues, with significantly higher levels in distant metastasis compared to primary tumors (**Figure 10B**, p<0.05). The ROC curve shows that ITGA11 significantly differentiates primary tumors from distant metastasis (**Figures 10C–E**). Furthermore, we achieved stable knockdown of ITGA11 in the human colon cancer cell line SW480 and subsequently evaluated the efficiency of this knockdown via Western blot analysis (**Figure 10F**). To assess the functional implications, we performed wound healing and transwell assays. The results of these assays demonstrated that ITGA11 knockdown significantly compromised the migratory ability of SW480 cells (**Figure 10A**; t - test, p < 0.05) and led to a substantial decrease in the number of invading cells (**Figure 10B**; t - test, p < 0.05). Collectively, these findings underscore the pivotal role of ITGA11 in the migration and invasion processes of CRC.

## Discussion

Approximately 20% of newly diagnosed CRC patients have synchronous distant metastasis and generally face a poor prognosis (26). While LNM signifies advanced disease, tumor cells may spread hematogenously before lymphatic metastasis. Previous reports indicate that about 18% of mCRC patients lack local lymph node involvement (27), and a novel CRC mouse model shows distant metastases can develop without prior lymph node involvement (9). Recurrence patterns in patients with CRLM undergoing liver transplantation without other metastasis suggest tumor cells may persist in circulation post-resection of primary and metastatic tumors. A more plausible explanation is that undetectable pre-operative metastasis account for most post-operative metastasis. Early hepatic metastases are often missed or undiagnosed by imaging, and by the time typical metastatic signs appear, radical surgery is usually no longer an option (28). Thus, identifying potential synchronous metastases or metastasis risks at early stage primary CRC is crucial (29).

The early occurrence of metastasis may stem from pre-existing, undetectable tumor dissemination prior to diagnosis or treatment. The primary tumor not only generates disseminated tumor cells but also establishes the pre-metastatic niche and modulates the immune response (30). Identifying the genetic traits of its stromal and extracellular matrix (ECM) components is vital for metastasis prediction (31). In cancer, EMT enables cancer cells to lose cell polarity and acquire a mesenchymal phenotype with enhanced stemness and migratory ability through complex interactions between fibers and proteins (13). Continuous remodeling of the ECM and actin cytoskeleton is closely associated with EMT, with integrins acting as physical linkers between the ECM and actin cytoskeleton, mediating mechanotransduction through interactions with major ECM components like collagen and fibronectin (31, 32). ITGA11, identified among LIMGs, shows the strongest correlation with EMT and high importance in various models predicting synchronous metastasis in CRC. ITGA11 promotes CAF invasion and CAF-induced tumor cell invasion, and associates with high-grade tumors and poor prognosis (33, 34). Mechanistically, ITGA11’s pro-invasive activity may stem from its ligand-dependent interaction with PDGFRβ, promoting downregulated JNK activation and ECM changes, including increased deposition of a strongly co-expressed pro-invasive stromal protein (tenascin-C, TNC) (35). PDGFRα+ ITGA11+ CAFs are associated with lymphovascular invasion (LVI) and early metastasis in early-stage bladder cancer, promoting lymphangiogenesis by recognizing the ITGA11 receptor SELE on lymphatic endothelial cells. Additionally, CHI3L1 from the CAF aligns the surrounding stroma to facilitate cancer cell intravasation and promote early tumor metastasis (36). Laminin LAMA5, a glycoprotein in the ECM, has been identified as a specific molecular target in mCRC (37). It is a key component of the vascular basement membrane, forming a scaffold for endothelial cell adhesion in conjunction with collagen IV, and is linked to the angiogenesis and tumor growth in CRLM (31, 38). Notably, LAMA5 exhibits the highest mutation frequency and CNVs in LIMGs, it is reported that genetic variant rs4925386 in chromosomal region 20q13.3 (LAMA5) significantly associated with CRC susceptibility (OR=0.93) (39). Periostin (POSTN), secreted by CAFs, accelerates angiogenesis, tumor invasion, and EMT via integrin interaction (40). Aberrant POSTN expression in CRC correlates strongly with peritoneal and distant organ metastasis. Meanwhile, POSTN+ CAFs significantly promote CRC cell migration and proliferation through hypoxia induced POSTN expression and secretion (41). The cbl-associated protein (CAP), encoded by the sorbin and SH3 domain-containing 1 (SORBS1) gene, plays a role in actin cytoskeleton regulation, receptor tyrosine kinase signaling, and cell adhesion. Overexpression of SORBS1 inhibits the PI3K/AKT pathway, blocks EMT, and promotes M1 macrophage polarization (42). Conversely, SORBS1 silencing accelerates EMT, boosts Filopodium-like Protrusion (FLP) formation via JNK/c-Jun activation in cancer cells, and elevates chemosensitivity by enhancing p53 protein accumulation (43). Nidogen1 (NID1), directly induced by SNAIL/SNAI-1 transcription factor, promotes EMT. It connects laminin, collagen, and proteoglycans to cell receptors, regulating cell polarization, migration, and invasion (44). Actin gamma 2 (ACTG2) is aberrantly expressed in cancers (45), with low levels in CRC, its overexpression inhibits CRC cell proliferation, migration, and invasion (46). Thrombospondin-1 (THBS1) inhibits angiogenesis and immune activity (47), but has complex, contradictory roles in carcinogenesis. THBS1 expression correlates with CRC mesenchymal phenotype, immunosuppression, and poor prognosis, promoting metastasis by exhausting cytotoxic T cells and impairing angiogenesis, especially at metastatic sites (48).

Pathological analysis of early-stage CRC aids in risk identification and treatment guidance. Factors like T4 stage, poor differentiation, intestinal perforation, lymphovascular/perineural invasion, inadequate lymph node examined, and positive surgical margins heighten disease progression risk (49). Our study revealed no significant LIMG score differences in T stage, vascular invasion, and histological type. However, a higher LIMG score correlated with tumor location, MSI status, lower KRAS mutation frequency, and lower TMB. Study shows that synchronous CRLM exhibit poorer prognosis and biological traits than metachronous ones (2, 50), with synchronous CRLM showing lower TMB (51). Moreover, patients with LNM- mCRC typically have fewer high-risk pathological features than LNM+ mCRC (52), indicating clinicopathological factors may inadequately assess the lymph node-independent metastasis in CRC, potentially leading to misdiagnosis. LIMG Score variations across primary tumor sites may stem from tumor site-genomic alteration correlations in mCRC. Left-sided CRC is more prone to synchronous liver metastases (7.1% vs. 11.6%), which may be anatomically influenced by venous shunting (53). Molecularly, right-sided primary tumor-derived MSS-type mCRC has a higher median TMB, with oncogenic alterations like KRAS, BRAF, and PIK3CA enriched, while APC and TP53 are more enriched in left-sided tumors (54).

Immunotherapy benefits CRC patients but is limited by the complex immunosuppressive TME and tumor heterogeneity (55). Our study found that high-risk patients have lower infiltration of anti-tumor immune cells (memory B cells, plasma cells, CD4+ T cells, NK cells, DCs), while exhibiting higher levels of M2-type TAMs that promote tumor growth and immunosuppression. Plasma cells, as terminal effector B cells, eliminate tumor cells via antibody-dependent cell-mediated cytotoxicity (ADCC) (56), forming an immunological chain with DCs and participating in tertiary lymphoid structures (TLS) formation (57). Reduced plasma cell and DC infiltration may indicate weakened antibody-mediated anti-tumor effects, TLS deficiency, and inadequate immune surveillance, potentially with increased Bregs or M2-type TAMs, leading to insufficient CD8^+^ T cell activation, further exacerbating immune escape, and diminished immunotherapy response.

Furthermore, we have identified ITGA11 as a critical factor in lymph node-independent metastasis in CRC, though its precise mechanism remains unclear. Our study demonstrated that knocking down ITGA11 significantly inhibits CRC cell migration and invasion. The mechanism behind ITGA11’s involvement in lymph node-independent synchronous metastasis may encompass multiple pathways, with EMT being a potential key player, which we aim to explore further.

Study limitations are several. A notable drawback is the small sample size, stemming from our single - center study and the scarcity of specimens meeting our criteria. This challenge weakened our study’s statistical power and robustness. Also, the heterogeneity of extensive stage II disease (N0), encompassing tumors confined to the serosa (T3) and those extending beyond it (T4), representing diverse histopathological risks. Additionally, even when an adequate number of lymph nodes are examined, there is a possibility of lymph node micrometastasis, as conventional histopathological examination cannot detect the presence of isolated tumor cells (ITCs) or micrometastases (MMs) within regional lymph nodes, and we did not perform ultra-staging for all these cases. Despite the limitations, we are committed to promoting multi - center, large - sample studies and employing multi - omics analysis in future research to better uncover the mechanisms underlying lymph node - independent distant metastasis and offer more reliable insights.

In summary, we integrated proteomics, multi-omics analysis, and machine learning to identify molecular features and developed an LIMGs signature based on nine genes, effectively predicting synchronous distant metastasis risk in stage I-II CRC patients. We also analyzed associations between the LIMG Score and pathological features, immune microenvironment and activity, and drug responses, offering insights into precise stratification and personalized therapy for CRC. Our findings also position ITGA11 as a crucial prognostic indicator for CRC metastasis.

## Data Availability

The datasets presented in this study can be found in online repositories. The names of the repository/repositories and accession number(s) can be found in the article/**Supplementary Material**.
