# Supplementary material for: Integrating proteomics and machine learning reveals characteristics and risks of lymph node-independent distant metastasis in colorectal cancer
Source: Front Immunol. 2025 Jul 21;16:1622528. doi: 10.3389/fimmu.2025.1622528 (PMC12322899; doi:10.3389/fimmu.2025.1622528)

Integrating Proteomics and Machine Learning Reveals Characteristics and Risks of Lymph Node-independent Distant Metastasis in Colorectal Cancer

Supplementary Material

Supplementary Figure S1：

GO and KEGG enrichment analysis of differentially expressed proteins among normal tissues vs. primary tumors, and distant metastases.

**A. GO_CC/BP/MF enrichment of differentially expressed proteins (Normal tissues vs. Primary Tumors)**


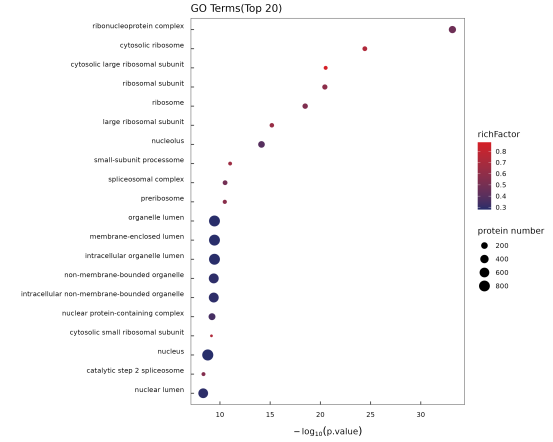

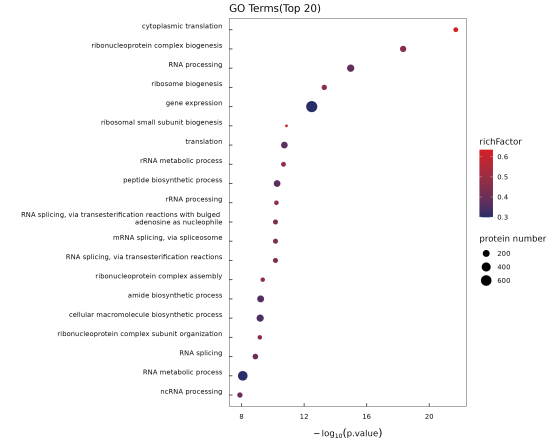

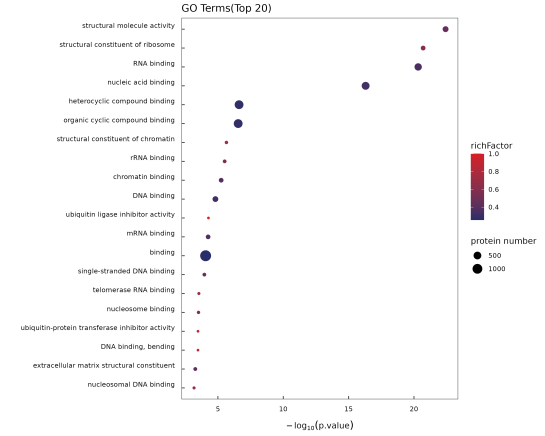


**B. KEGG enrichment of differentially expressed proteins (Normal tissues vs. Primary Tumors)**


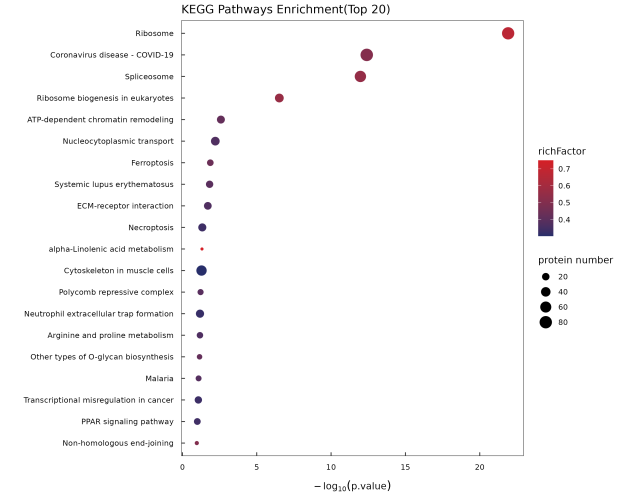


**C. GO_CC/BP/MF enrichment of differentially expressed proteins (Normal vs. Distant Metastases)**


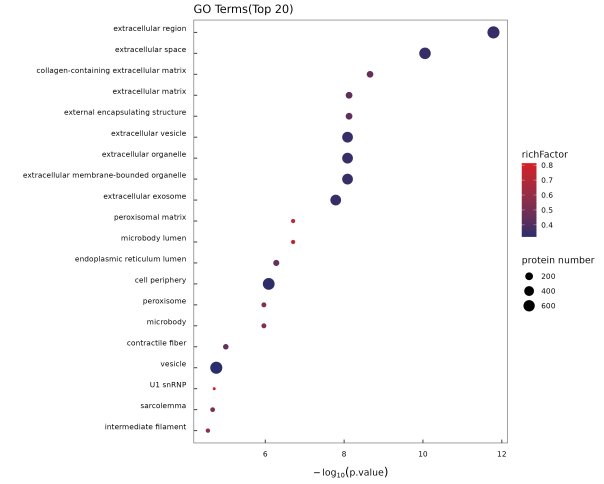

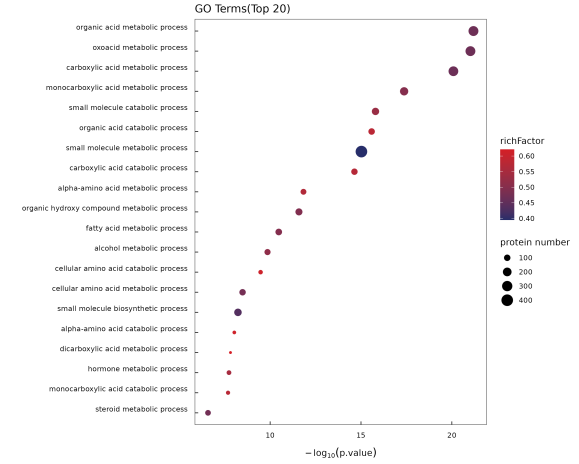

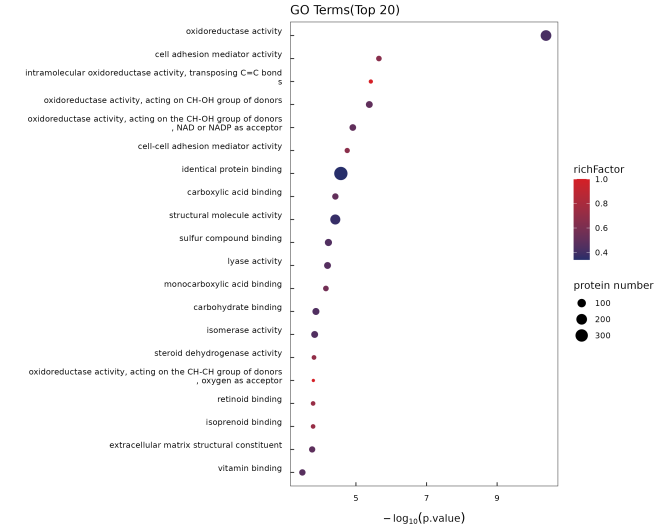


**D. KEGG enrichment of differentially expressed proteins (Normal vs. Tumor)**


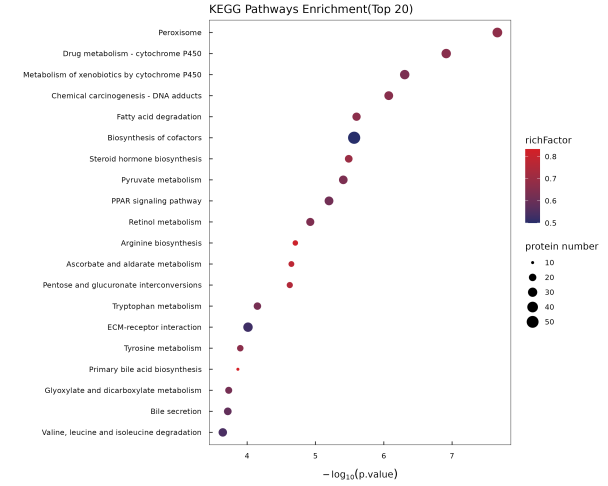


Supplementary Figure S2：

The enrichment analysis results of other 4 modules (1,3,4,5) identified by MCODE in the PPI network using Cytoscape.

1. **5 modules identified by MCODE in Cytoscape:**

**
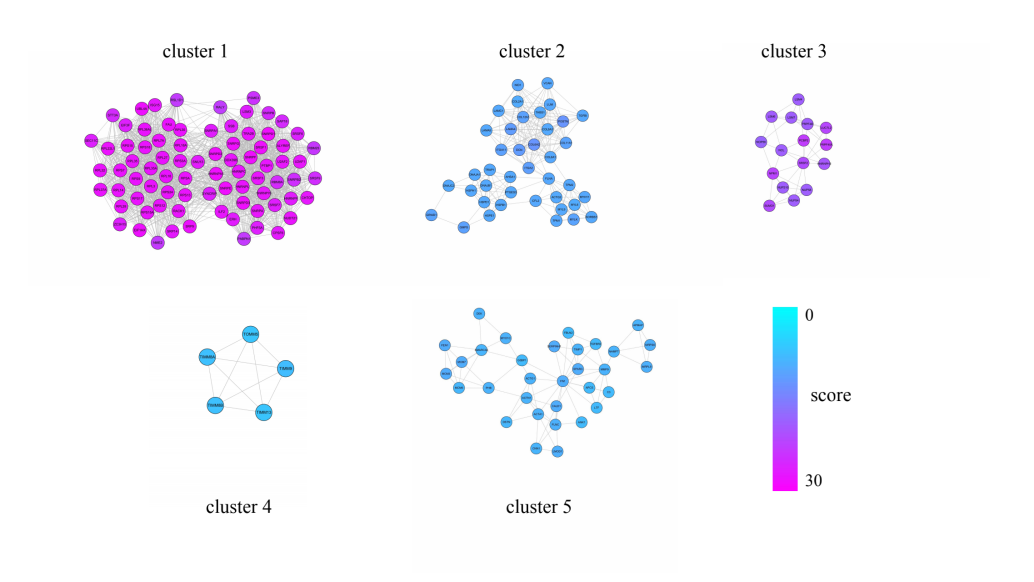
**

**B. GO enrichment of cluster1:**


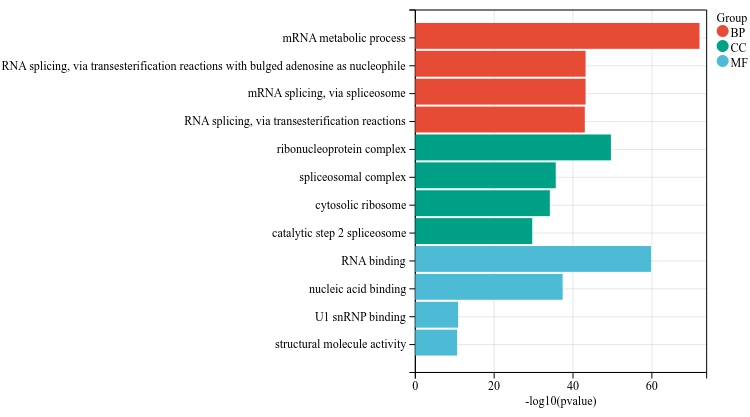


**C. KEGG enrichment of cluster1:**


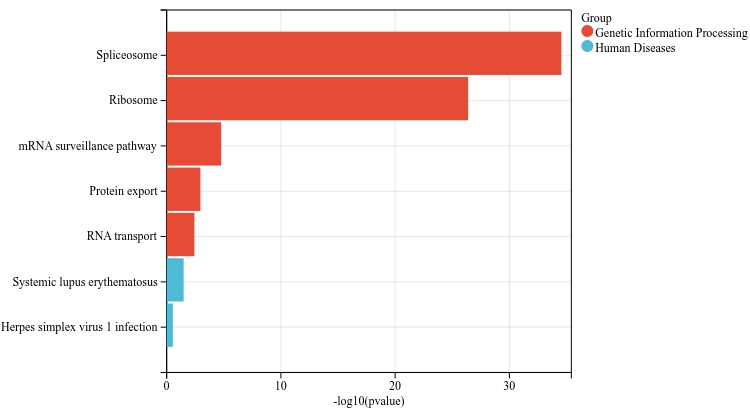


**D. GO enrichment of cluster3:**

**
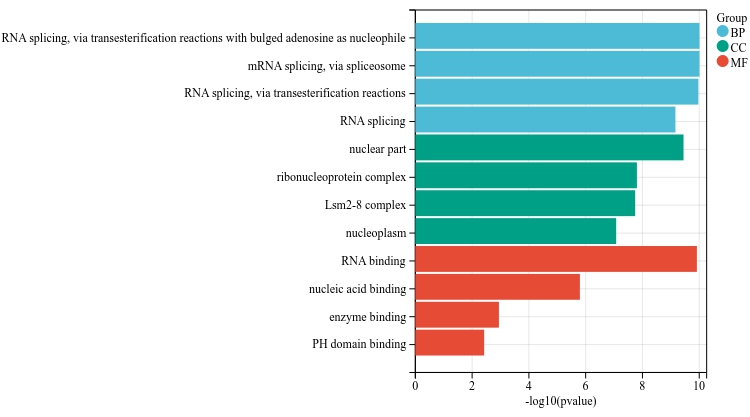
**

**E. KEGG enrichment of cluster3:**


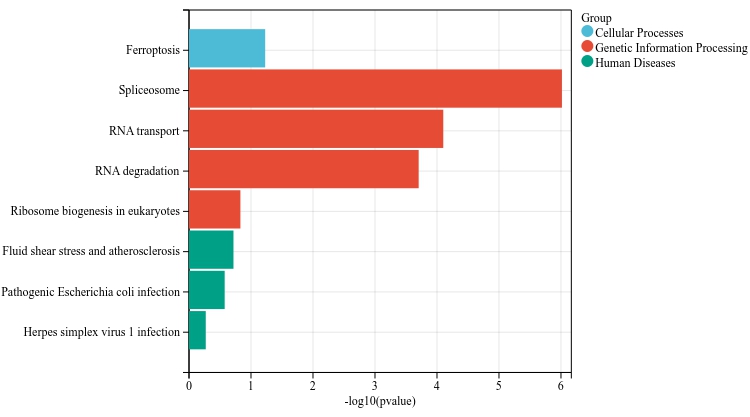


**F. GO enrichment of cluster4:**


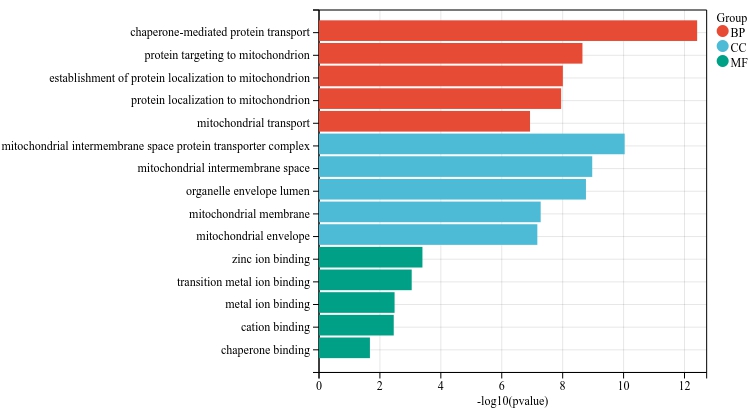


**G. GO enrichment of cluster5:**


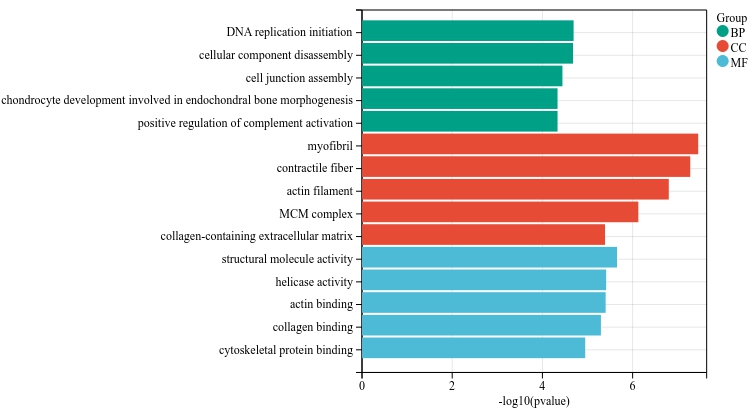


**H. KEGG enrichment of cluster5:**

**
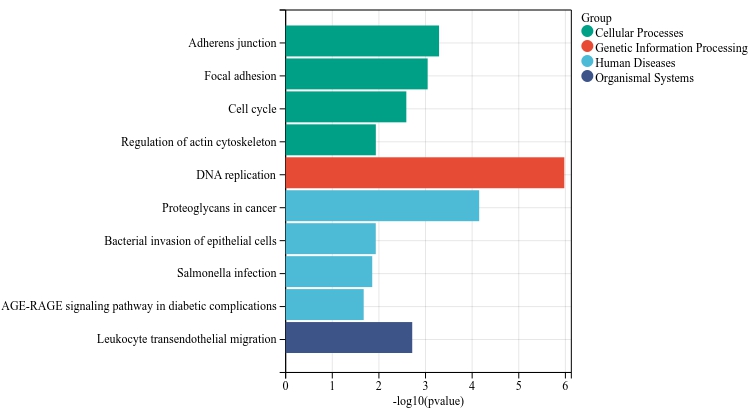
**

Supplementary Figure S3：

The associations between the RiskScore and MMR/KRAS/BRAF/TP53 status.

.

**A: Associations between the LIMG Score and age.**


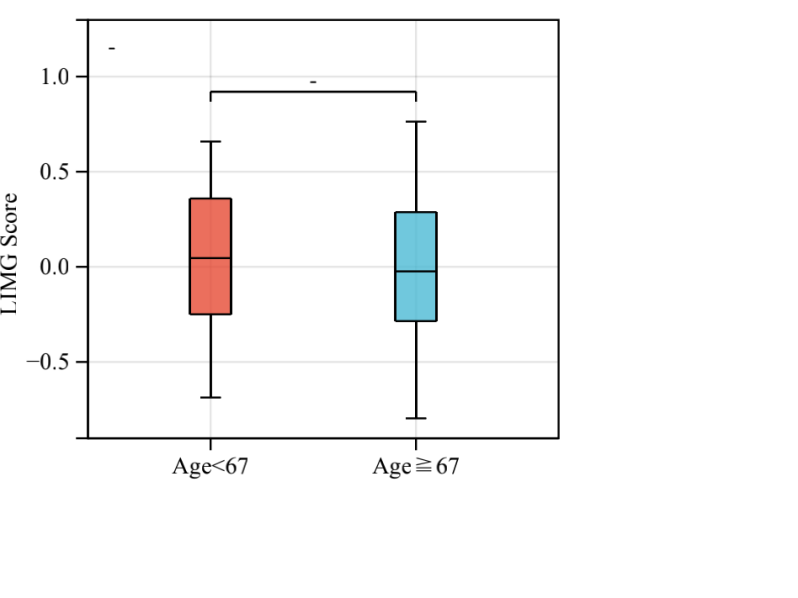


**B: Associations between the LIMG Score and sex.**

**
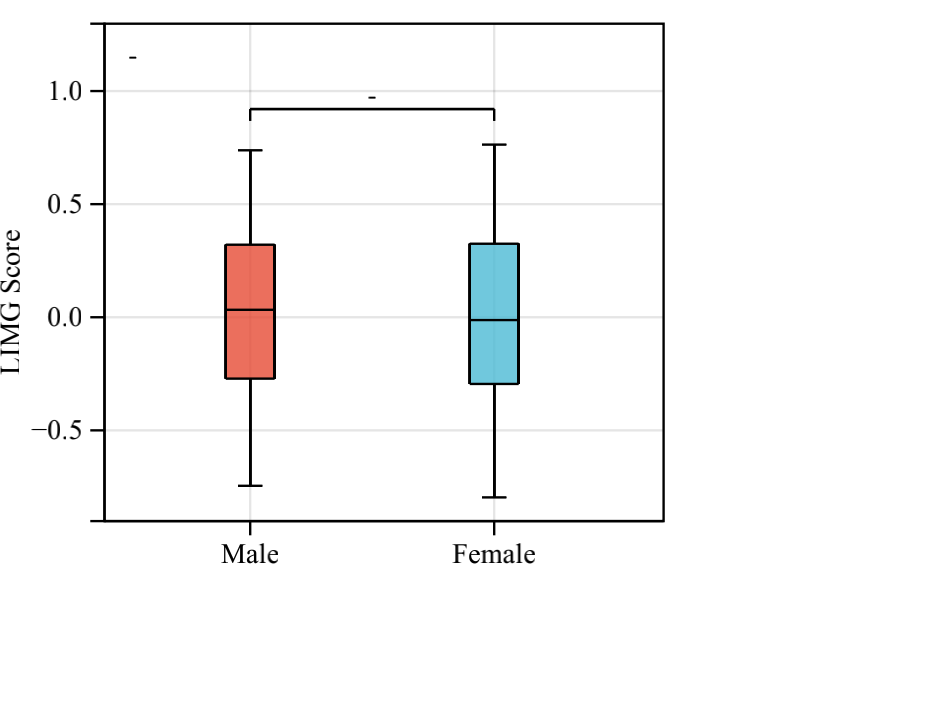
**

**C: Associations between the LIMG Score and vascular invasion.**

**
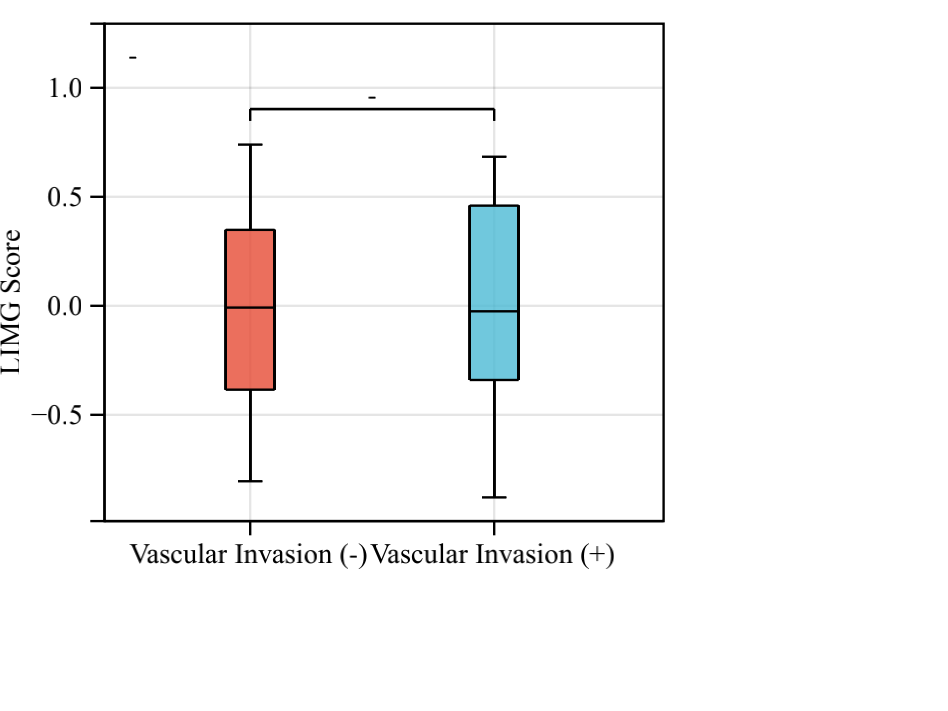
**

**D: Associations between the LIMG Score and histological type.**

**
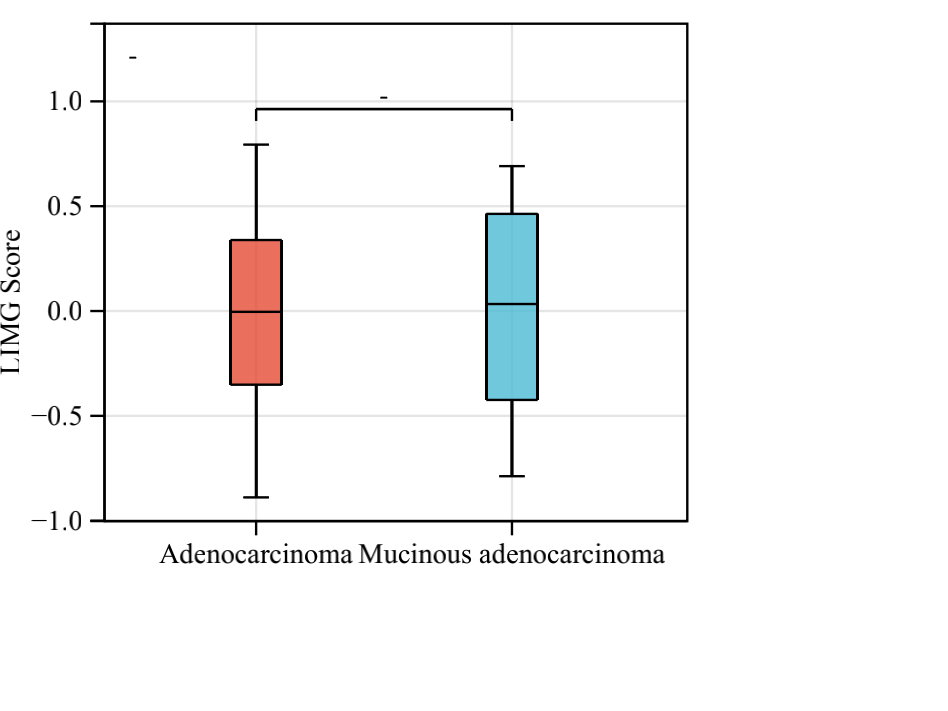
**

**E: Associations between the LIMG Score and BRAF mutation.**

**
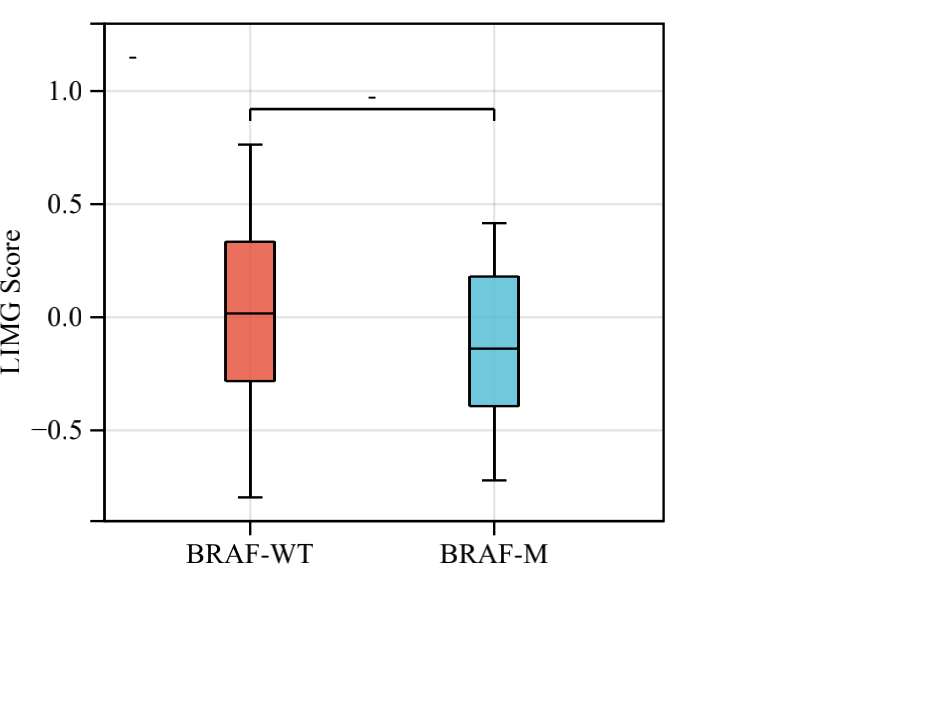
**

**F: Associations between the LIMG Score and TP53 mutation.**

**
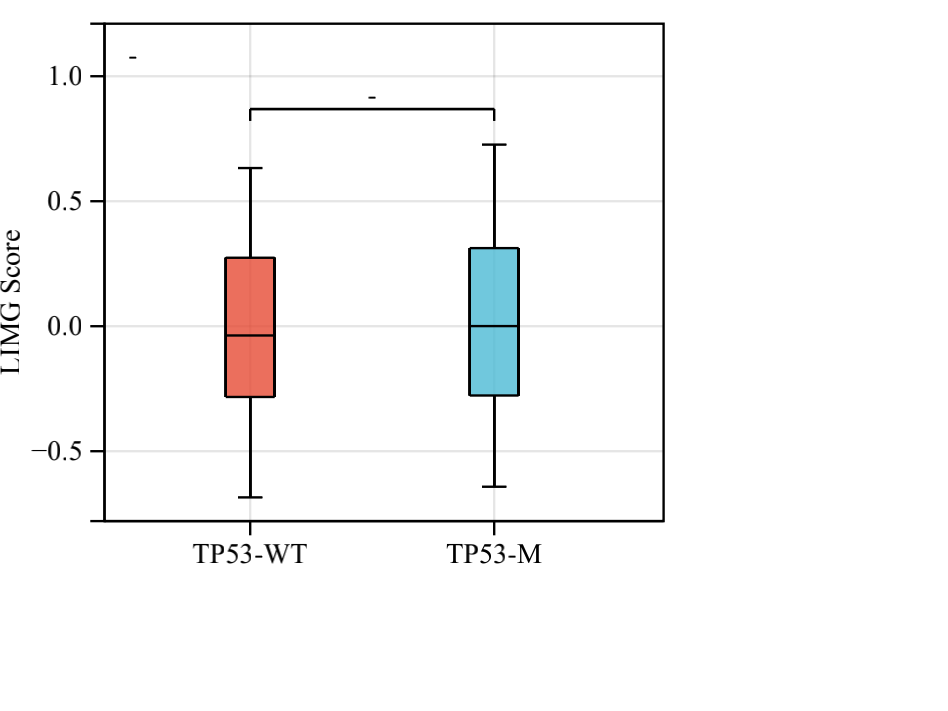
**

Supplementary Figure S4：

The results of GO and KEGG enrichment analyses of LIMGs.

**A: GO enrichment of TDRGs**

**
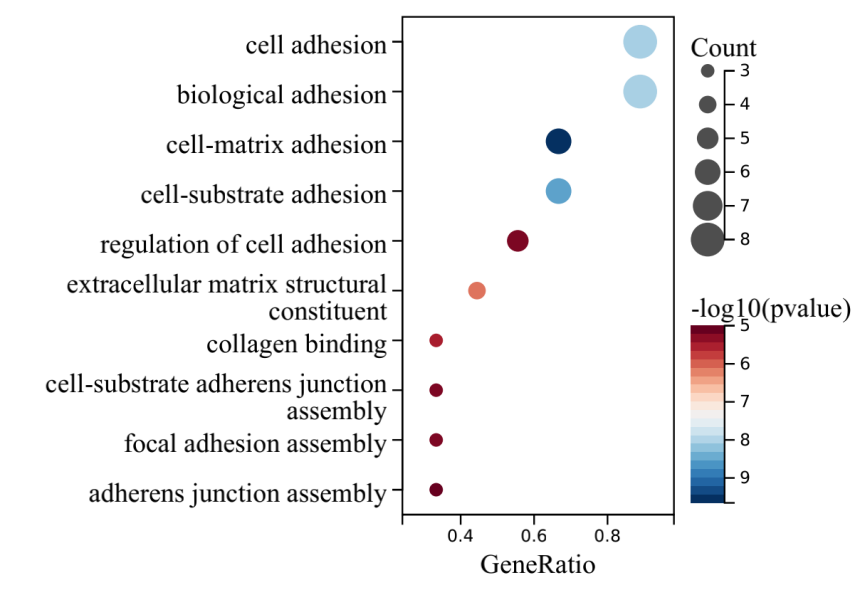
**

**B: KEGG enrichment of TDRGs**

**
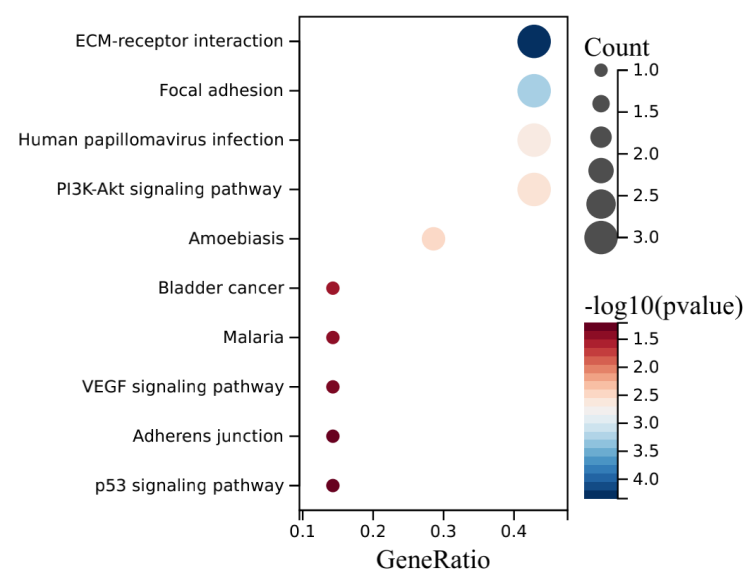
**

Supplementary Figure S5：

The Protein expression patterns of 9 LIMGs in primary tumors, and distant metastases.


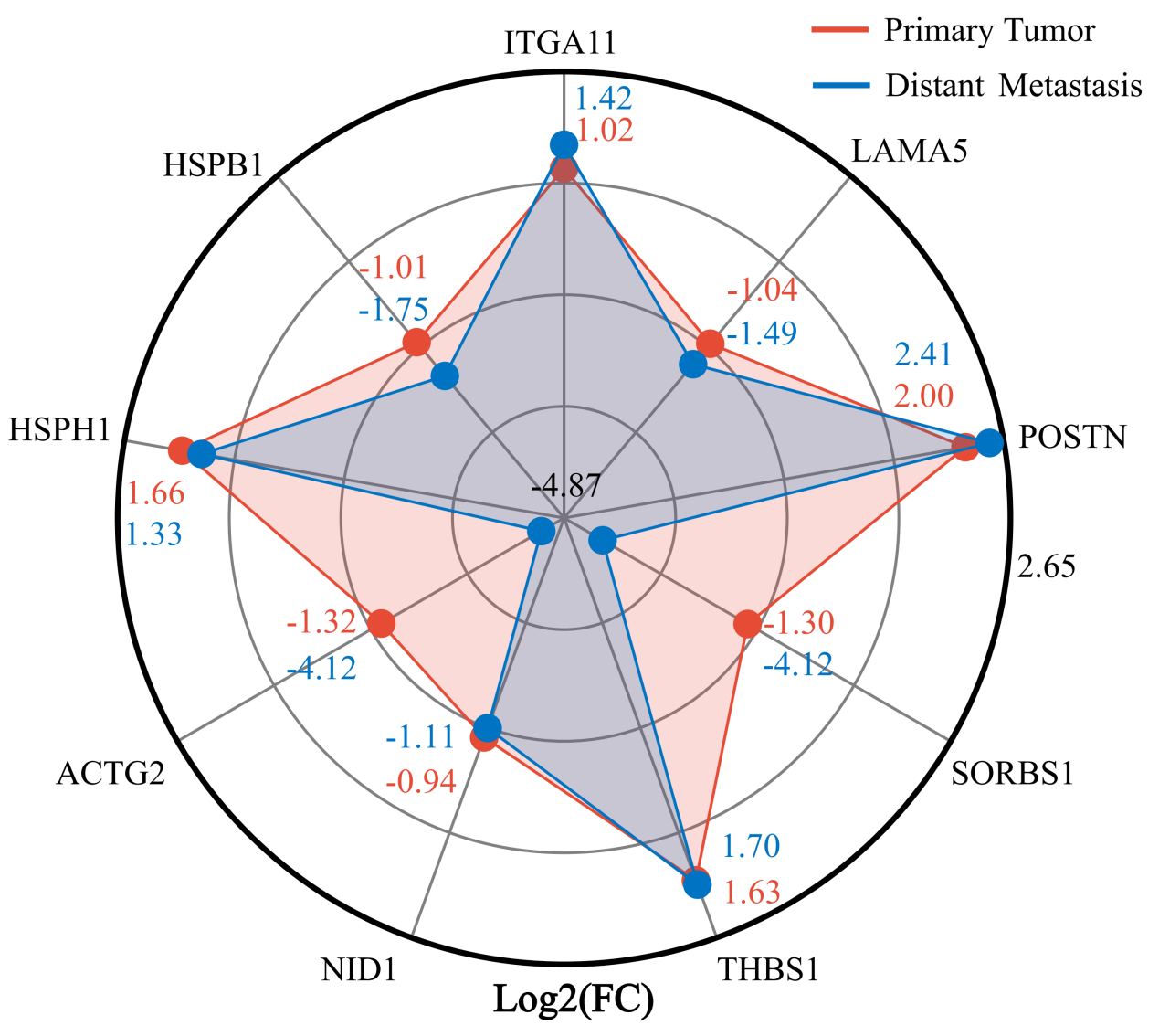

Supplement: Supplementary file 2 [file DataSheet1.docx]
